# Artificial Intelligence Techniques That May Be Applied to Primary Care Data to Facilitate Earlier Diagnosis of Cancer: Systematic Review

**DOI:** 10.2196/23483

**Published:** 2021-03-03

**Authors:** Owain T Jones, Natalia Calanzani, Smiji Saji, Stephen W Duffy, Jon Emery, Willie Hamilton, Hardeep Singh, Niek J de Wit, Fiona M Walter

**Affiliations:** 1 Primary Care Unit Department of Public Health & Primary Care University of Cambridge Cambridge United Kingdom; 2 Wolfson Institute for Preventive Medicine Queen Mary University of London London United Kingdom; 3 Centre for Cancer Research and Department of General Practice University of Melbourne Victoria Australia; 4 College of Medicine and Health University of Exeter Exeter United Kingdom; 5 Center for Innovations in Quality, Effectiveness and Safety Michael E DeBakey Veterans Affairs Medical Center and Baylor College of Medicine Houston, TX United States; 6 Julius Center for Health Sciences and Primary Care UMC Utrecht Utrecht Netherlands

**Keywords:** artificial intelligence, machine learning, electronic health records, primary health care, early detection of cancer

## Abstract

**Background:**

More than 17 million people worldwide, including 360,000 people in the United Kingdom, were diagnosed with cancer in 2018. Cancer prognosis and disease burden are highly dependent on the disease stage at diagnosis. Most people diagnosed with cancer first present in primary care settings, where improved assessment of the (often vague) presenting symptoms of cancer could lead to earlier detection and improved outcomes for patients. There is accumulating evidence that artificial intelligence (AI) can assist clinicians in making better clinical decisions in some areas of health care.

**Objective:**

This study aimed to systematically review AI techniques that may facilitate earlier diagnosis of cancer and could be applied to primary care electronic health record (EHR) data. The quality of the evidence, the phase of development the AI techniques have reached, the gaps that exist in the evidence, and the potential for use in primary care were evaluated.

**Methods:**

We searched MEDLINE, Embase, SCOPUS, and Web of Science databases from January 01, 2000, to June 11, 2019, and included all studies providing evidence for the accuracy or effectiveness of applying AI techniques for the early detection of cancer, which may be applicable to primary care EHRs. We included all study designs in all settings and languages. These searches were extended through a scoping review of AI-based commercial technologies. The main outcomes assessed were measures of diagnostic accuracy for cancer.

**Results:**

We identified 10,456 studies; 16 studies met the inclusion criteria, representing the data of 3,862,910 patients. A total of 13 studies described the initial development and testing of AI algorithms, and 3 studies described the validation of an AI algorithm in independent data sets. One study was based on prospectively collected data; only 3 studies were based on primary care data. We found no data on implementation barriers or cost-effectiveness. Risk of bias assessment highlighted a wide range of study quality. The additional scoping review of commercial AI technologies identified 21 technologies, only 1 meeting our inclusion criteria. Meta-analysis was not undertaken because of the heterogeneity of AI modalities, data set characteristics, and outcome measures.

**Conclusions:**

AI techniques have been applied to EHR-type data to facilitate early diagnosis of cancer, but their use in primary care settings is still at an early stage of maturity. Further evidence is needed on their performance using primary care data, implementation barriers, and cost-effectiveness before widespread adoption into routine primary care clinical practice can be recommended.

## Introduction

### Background

Cancer control is a global health priority, with 17 million new cases diagnosed worldwide in 2018. In high-income countries such as the United Kingdom, approximately half the population over the age of 50 years will be diagnosed with cancer in their lifetime [1]. Although the National Health Service (NHS) currently spends approximately £1 billion (US $1.37 billion) on cancer diagnostics per year [2], the United Kingdom lags behind comparable European nations with their cancer survival rates [3].

In gatekeeper health care systems such as the United Kingdom, most people diagnosed with cancer first present in primary care [4], where general practitioners evaluate (often vague) presenting symptoms and decide on an appropriate management strategy, including investigations, specialist referral, or reassurance. More accurate assessment of these symptoms, especially for patients with multiple consultations, could lead to earlier diagnosis of cancer and improved outcomes for patients, including improved survival rates [5,6].

There is accumulating evidence that artificial intelligence (AI) can assist clinicians in making better clinical decisions or even replace human judgment, in certain areas of health care. This is due to the increasing availability of health care data and the rapid development of big data analytic methods. There has been increasing interest in the application of AI in medical diagnosis, including machine learning and automated analysis approaches. Recent studies have applied AI to patient symptoms to improve diagnosis [7,8], to retinal images for the diagnosis of diabetic retinopathy [9], to mammography images for breast cancer diagnosis [10,11], to computed tomography (CT) scans for the diagnosis of intracranial hemorrhages [12], and to images of blood films for the diagnosis of acute lymphoblastic leukemia [13].

Few AI techniques are currently implemented in routine clinical care. This may be due to uncertainty over the suitability of current regulations to assess the safety and efficacy of AI systems [14-16], a lack of evidence about the cost-effectiveness and acceptability of AI systems [14], challenges to implementation into existing electronic health records (EHRs) and routine clinical care, and uncertainty over the ethics of using AI systems. A recent review of AI and primary care reported that research on AI for primary care is at an early stage of maturity [17], although research on AI-driven tools such as symptom checkers for patient and clinical users are more mature [18-21].

**Figure 1:**
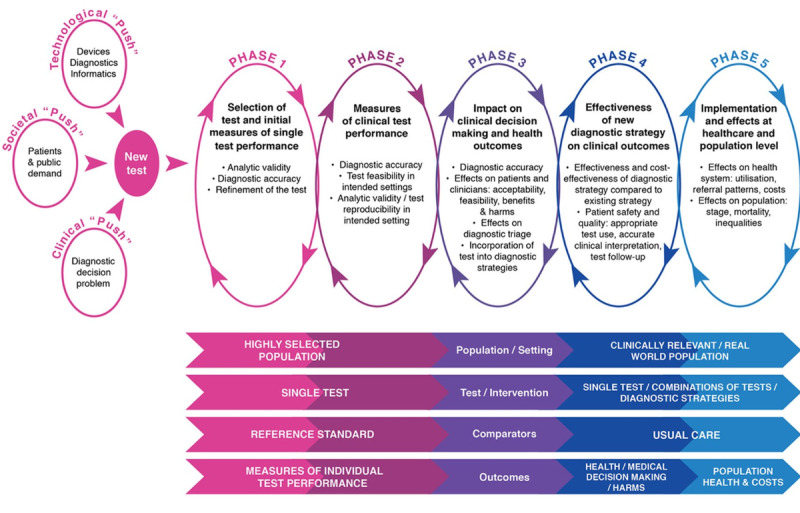
The CanTest Framework [22].

The CanTest framework [22] (Figure 1) establishes the developmental phases required to ensure that new diagnostic tests or technologies are fit for purpose when introduced into clinical practice. It provides a roadmap for developers and policy makers to bridge the gap from the development of a diagnostic test or technology to its successful implementation. We used this framework to guide the assessment of the studies identified in this review.

### Objectives

Few studies of AI-based techniques for the early detection of cancer have been undertaken in primary care settings [17]. Therefore, the aim of this systematic review is to identify AI techniques that facilitate the early detection of cancer and could be applied to primary care EHR data. We also aim to summarize the diagnostic accuracy measures used to evaluate existing studies and evaluate the quality of the evidence, the phase of development the AI technologies have reached, the gaps that exist in the evidence, and the potential for use in primary care. As many commercial technological developments are not documented in academic publications, we also performed a parallel scoping review of commercially available AI-based technologies for the early detection of cancer that may be suitable for implementation in primary care settings.

## Methods

### Search Strategy and Selection Criteria

This study was conducted in accordance with PRISMA (Preferred Reporting Items for Systematic Reviews and Meta-analysis) guidelines [23], and the protocol was registered with PROSPERO (an international prospective register of systematic reviews) before conducting the review (CRD42020176674) [24]. All aspects of the protocol were reviewed by the senior research team.

We included all primary research articles published in peer-reviewed journals, without language restrictions, from January 01, 2000, to June 11, 2019. Studies were included if they provided evidence around the accuracy, utility, acceptability, or cost-effectiveness of applying AI techniques to facilitate the early detection of cancer and could be applied to primary care EHRs (ie, to the types of data found in primary care EHRs) [22]. We included AI techniques based on any type of data that were relevant to primary care settings, including coded data and free text. We included all types of study design, as we anticipated that there would be few relevant randomized controlled trials. We kept our search terms broad to not miss relevant studies and carefully considered evidence from any health care system to assess whether the evidence could be applied to primary care settings.

As our aim is to identify AI techniques that would be applicable in primary care clinical settings, we excluded studies that incorporated data not typically available in primary care EHRs in the early diagnostic stages (eg, histopathology images, magnetic resonance imaging, or CT scan images). We also excluded studies that only described the development of an AI technique without any testing or evaluation data, studies that did not incorporate an element of machine learning (ie, with training and testing or validation steps), studies that used AI techniques for biomarker discovery alone, and studies that were based on sample sizes of less than 50 cases or controls. Machine learning techniques and neural networks have been described since the 1960s [25,26]; however, they were initially limited by computing power and data availability. We chose to start our search in 2000, as this was when the earliest research describing the new wave of machine learning techniques emerged [27].

We searched MEDLINE, Embase, SCOPUS, and Web of Science bibliographic databases, using keywords related to AI, cancer, and early detection. We extended these systematic searches through manual searching of the reference lists of the included studies. We contacted study authors, where required. Where studies were not published in English, we identified suitably qualified native speakers to help assess these studies. We performed a parallel scoping review to look for commercially developed AI technologies that were not identified through systematic searches, thus unpublished and not scientifically evaluated. This included manually searching commercial research archives and networks (eg, arXiv [28], Google [29], Microsoft [30], and IBM [31]), reviewing the computer-based technologies identified in 3 recent reviews [19-21], and manually searching for further technologies mentioned in the text or references of the studies and websites included in these reviews.

Following duplicate removal, 1 author (OJ) screened titles and abstracts to identify studies that fit the inclusion criteria. Of the titles and abstracts, 17.42% (1838/10,456) were checked by 2 other authors (SS and NC); interrater reliability was excellent at 96.24% (1769/1838). Any disagreements were discussed by the core research team (OJ, SS, NC, and FW), and a consensus was reached. Three reviewers (OJ, SS, and NC) independently assessed the full-text articles for inclusion in the review. Any disagreements were resolved by a consensus-based decision.

### Data Analysis

Data extraction was undertaken independently by at least two reviewers (OJ, SS, and NC) into a predesigned data extraction spreadsheet. The research team met regularly to reach consensus by discussing and resolving any differences in data extraction. One author (OJ) amalgamated the data extraction spreadsheets, summarizing the data where possible.

The main summary measures collected included sensitivity, specificity, positive predictive value (PPV), negative predictive value (NPV), area under the receiver operating characteristic (AUROC) curve, and any other diagnostic accuracy measures of the AI techniques. Secondary outcomes include the types of AI used, the type of data used to train and test the algorithms, and how these algorithms were evaluated. We also collected data, where identified, on cost-effectiveness and patient or clinician acceptability.

Risk of bias assessment was undertaken for all full-text papers by 2 independent researchers (OJ and NC) using the quality assessment of diagnostic accuracy studies-2 (QUADAS-2) critical appraisal tool [32]. OJ assessed all studies, and 50% (40/79) of them were cross-checked by NC. Any disagreements in the assessment were resolved by consensus discussion.

The studies identified were heterogeneous, employing various AI techniques and using different outcome measures for evaluation. Hence, a meta-analysis of the data was not possible, and we chose to use a narrative synthesis approach, following established guidance on its methodology [33]. We aimed to summarize the findings of the identified studies using primarily a textual approach, while also providing an overview of the quantitative outcome measures used in the studies. Once data extraction was completed, we explored the relationships that emerged within the data.

Full details of our review question, search strategy, inclusion or exclusion criteria, and data extraction methodology are described in Multimedia Appendices 1 [1-5,7-9,11-13,34-38] and 2, and the full list of excluded studies is provided in Multimedia Appendix 3 [34,39-114].

## Results

**Figure 2:**
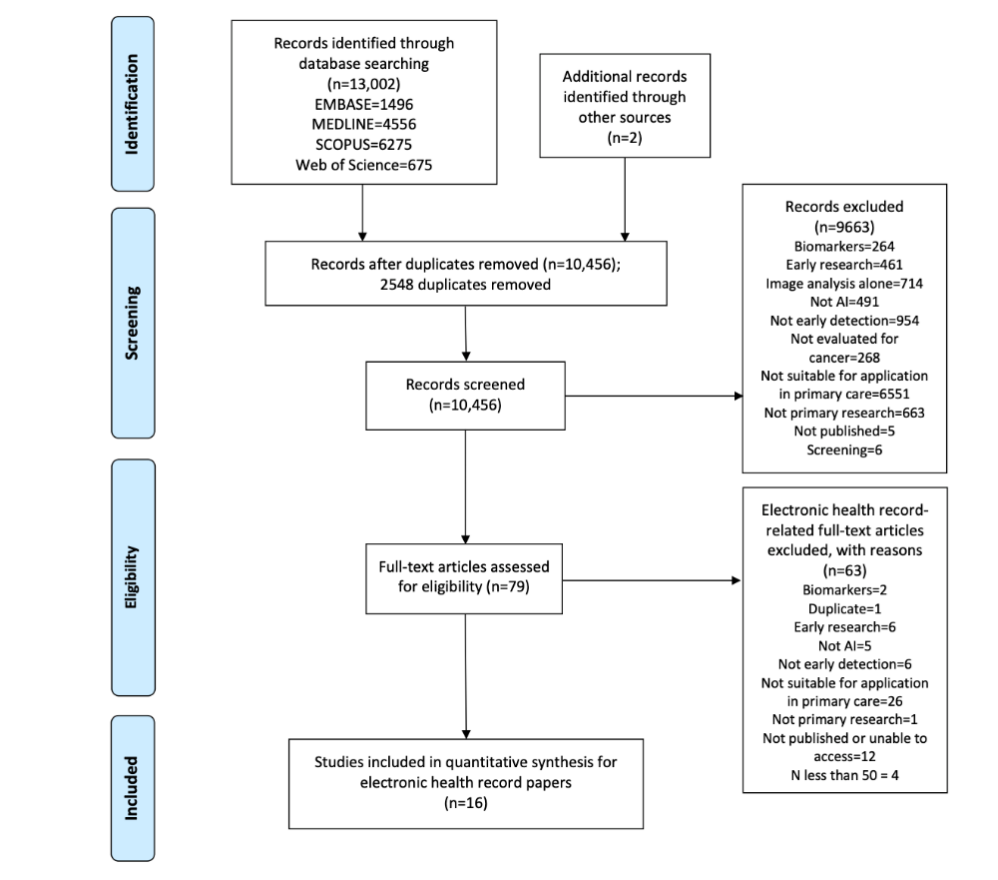
PRISMA (Preferred Reporting Items for Systematic Reviews and Meta-analysis) flow diagram for studies included in the review. AI: artificial intelligence.

A total of 13,004 articles were identified in database searches (including 2548 duplicates), and 793 articles underwent full-text review. Of the 79 articles that were related to EHRs, 16 met the inclusion criteria and were included in this analysis (Figure 2), representing the data of 3,862,910 patients. No articles identified through other sources or reference lists met the inclusion criteria.

Tables 1 and 2 show the main study characteristics for the 16 included studies, including the modality of AI used. Supplementary information on the variables included in the AI techniques is available in Multimedia Appendix 4 [34,39-53]. We categorized the variables included into the following categories: demographics, symptoms, comorbidities, lifestyle history, examination findings, blood results, and other. Most studies (n=13) described the initial development and testing of an AI technique [39-51]. Three studies validated the AI technique developed by Kinar et al [48] in independent data sets from 3 different countries (Israel, United States, and United Kingdom) [34,52,53].

**Table 1:** Study details including modality of artificial intelligence and adopted comparison or control.

Study		Authors’ origin	Cancer	Modality of artificial intelligence	Comparison or control			
					Histopathology	Specialist	Not stated	Other
**Development studies**								
	Alzubi et al, 2019 [39]	Jordan and India	Lung cancer	WONN-MLB^a^	X^b^	—^c^	—	1^d^
	Chang et al, 2009 [40]	Taiwan	Pancreatic Cancer	BPNN^e^; LR^f^	—	—	X	2^g^; 3^h^
	Cooper et al, 2018 [41]	United Kingdom	Colorectal Cancer	ANN^i^; CVT^j^; LR	X	X	—	4^k^
	Cowley et al, 2013 [42]	United Kingdom	Colorectal Cancer	BPANN^l^	—	X	—	2; 5^m^
	Daqqa et al, 2017 [43]	Gaza, Palestine	Leukemia	SVM^n^; DT^o^; K-NN^p^	X	—	—	2
	Goryński et al, 2014 [44]	Poland	Lung cancer	MLP-ANN^q^	X	X	—	—
	Hart et al, 2018 [45]	United States	Lung cancer	BPANN	—	—	X	2; 6^r^
	Kalra et al, 2003 [46]	United States	Prostate cancer	BPNN	X	—	—	2; 3
	Kang et al, 2017 [47]	China	Any cancer	BPNN; CVT; SVM; DT	X	X	—	2
	Kinar et al, 2016 [48]	Israel and United States	Colorectal Cancer	DT/RF^s^; GBM^t^; CVT	X	X	—	3; 6
	Kop et al, 2016 [49]	The Netherlands	Colorectal Cancer	CART^u^; RF; LR; CVT	X	X	—	—
	Miotto et al, 2016 [50]	United States	Multiple diseases and cancers	DNN^v^; RF	—	X	—	2; 3
	Payandeh et al, 2009 [51]	Iran	CML^w^ and lymphoproliferative disorders	MLP-ANN	X	X	—	3
**Validation studies**								
	Birks et al, 2017 [52]	United Kingdom	Colorectal Cancer	DT/RF; GBM; CVT	X	X	—	—
	Hornbrook et al, 2017 [34]	United States	Colorectal Cancer	DT/RF; GBM; CVT	X	X	—	—
	Kinar et al, 2017 [53]	Israel	Colorectal Cancer	DT/RF; GBM; CVT	X	X	—	—

^a^WONN-MLB: weight optimized neural network with maximum likelihood boosting.

^b^X: corresponding control used in this study.

^c^Not used in this study.

^d^1: previously developed artificial intelligence methods.

^e^BPNN: back propagation neural network.

^f^LR: logistic regression.

^g^2: other artificial intelligence methods developed by this author.

^h^3: other statistical (ie, non-artificial intelligence) techniques.

^i^ANN: artificial neural network.

^j^CVT: cross-validation techniques.

^k^4: colonoscopy.

^l^BPANN: back propagation artificial neural network.

^m^5: primary care clinicians.

^n^SVM: support vector machine.

^o^DT: decision tree.

^p^K-NN: K-nearest neighbor.

^q^MLP-ANN: multilayer perceptron artificial neural network.

^r^6: screening tests (eg, low-dose computed tomography scan and fecal occult blood test).

^s^RF: random forest.

^t^GBM: gradient boosting model.

^u^CART: classification and regression trees.

^v^DNN: deep neural network.

^w^CML: chronic myeloid leukemia.

The study authors originated from a variety of countries, including the United States (n=5), countries in the Middle East (n=5), Europe (n=5), and Asia (n=3), with some studies involving multiple countries. The AI techniques were most commonly developed to identify colorectal cancer (n=7) [34,41,42,48,49,52,53], although they also addressed lung cancer (n=3) [39,44,45], hematological cancers (n=2) [43,51], pancreatic cancer (n=1) [40], prostate cancer (n=1) [46], and multiple cancers (n=2) [47,50].

Neural networks were the dominant technique employed (n=10) [39-42,44-47,50,51], with many neural network subtypes mentioned. The study by Miotto et al [50] was the only study to include a processed form of the free text notes in the data used by the AI technique, although the work described by Kop et al [49] was developed in a subsequent study to include clinical free text data [115].

The majority of studies (n=9) used a combination of histopathological diagnoses and expert opinion as the control for their study [34,41,44,47-49,51-53]. The clinical control group was unclear in 2 studies [40,45]. Many studies used multiple AI techniques and then compared them with each other (n=8) [40,42,43,45-47,49,50]. Some studies used non-AI techniques, such as logistic regression and screening tests, as comparators for the performance of the AI technique that was being developed [40,41,45,46,48-51].

**Table 2:** Study details: patient variables.

Study		Patient variables									
		Age	Sex	Demographics	Symptoms	Comorbidities	Lifestyle	Examination	FBC^a^	Other blood tests	Other^b^
**Development studies**											
	Alzubi et al, 2019 [39]	X^c^	—^d^	—	X	X	X	—	—	—	X
	Chang et al, 2009 [40]	X	X	—	X	X	X	—	X	X	—
	Cooper et al, 2018 [41]	X	X	X	—	—	—	—	—	—	X
	Cowley et al, 2013 [42]	—	—	—	X	X	X	—	—	—	X
	Daqqa et al, 2017 [43]	—	—	—	—	—	—	—	X	—	—
	Goryński et al, 2014 [44]	X	X	X	X	X	X	X	X	X	X
	Hart et al, 2018 [45]	X	X	X	—	X	X	X	—	—	—
	Kalra et al, 2003 [46]	X	—	X	X	X	—	X	—	X	—
	Kang et al, 2017 [47]	X	X	—	—	—	—	X	X	X	X
	Kinar et al, 2016 [48]	X	X	—	—	—	—	—	X	—	—
	Kop et al, 2016 [49]	X	X	—	X	X	X	X	X	X	X
	Miotto et al, 2016 [50]	—	—	X	X	X	X	X	—	X	X
	Payandeh et al, 2009 [51]	—	—	—	—	—	—	—	X	—	—
**Validation studies**											
	Birks et al, 2017 [52]	X	X	—	—	—	—	—	X	—	—
	Hornbrook et al, 2017 [34]	X	X	—	—	—	—	—	X	—	—
	Kinar et al, 2017 [53]	X	X	—	—	—	—	—	X	—	—

^a^FBC: full blood count.

^b^More detail on other variables included is available in Multimedia Appendix 4.

^c^X: corresponding variable used in this study.

^d^Not used in this study.

Most of the studies (n=12) included blood test results, all suitable for use in primary care settings. Age was also commonly included (n=12). Other variables used were sex (n=10), demographics (n=5), symptoms (n=7), comorbidities (n=8), lifestyle history (n=7), examination findings (n=6), medication or prescription history (n=3), spirometry results (n=2), urine dipstick results (n=1), fecal immunochemical test results (n=1), x-ray text reports (n=1), and referrals (n=1).

Table 3 shows the study designs and populations. Most studies used data sets originating from specialist care settings (n=7) [39,40,42-44,46,51], with only 3 studies using solely primary care patient data [41,49,52]. Kinar et al [48] included a follow-up validation study based on the health improvement network (THIN) database, also using primary care data. Several studies used a mixture of primary and secondary care patient data (n=5) [34,47,48,50,53].

**Table 3:** Study population and study design.

Study details		Population from health care setting	Database used	Disease positive population (patients)	Disease negative population (patients)	Training set (patients)	Testing set (patients)
**Development studies**							
	Alzubi et al, 2019 [39]	Specialist care	Wroclaw Thoracic Surgery Centre	1200 in total; numbers of disease positive and negative unclear	1200 in total; numbers of disease positive and negative unclear	N/S^a^	1000
	Chang et al, 2009 [40]	Specialist care (unclear)	“a certain medical center”	194	157^b^	234	117
	Cooper et al, 2018 [41]	Primary care	NHS^c^ Bowel Cancer Screening Programme comparative study [116]	549	1261	N/S	N/S
	Cowley et al, 2013 [42]	Specialist care	2-week wait colorectal referrals to Castle Hill Hospital	74	703	777	100
	Daqqa et al, 2017 [43]	Specialist care	Complete Blood Count test repository, European Gaza Hospital	2000	2000	N/S	N/S
	Goryński et al, 2014 [44]	Specialist care	Patients treated at Kuyavia and Pomerania Centre of pulmonology	103	90	97	48
	Hart et al, 2018 [45]	Other (survey)	National Health Interview Survey	649	488,418	342,347	146,719
	Kalra et al, 2003 [46]	Specialist care	Men whose samples were tested at 6 sites in the United States^d^	348	N/S	218	144
	Kang et al, 2017 [47]	Mixed	Database of Ci Ming Health Checkup Center	650	1650	N/S	N/S
	Kinar et al, 2016 [48]^e^	Mixed	Maccabi Health Services EMRs^f^ linked to the Israel Cancer Registry	2437	463,670	466,107	139,205
	Kop et al, 2016 [49]	Primary care	6 anonymized data sets from 3 urban regions, each covering a GP^g^ recording system	1292	263,879	N/S	N/S
	Miotto et al, 2016 [50]	Mixed	Mount Sinai Data Warehouse	276,214 patients with 78 diseases	276,214 patients with 78 diseases	200,000	76,214
	Payandeh et al, 2009 [51]	Specialist care	Blood test results from patients at the Taleghani Hospital	450	N/S	360	132
**Validation studies**							
	Birks J et al, 2017 [52]	Primary care	Clinical Practice Research Datalink	5141	2,220,108	N/A^h^	N/A
	Hornbrook et al, 2017 [34]	Mixed	Kaiser Permanente North West EHR^i^ system, Kaiser Permanente Tumor Registry	900	16,195	N/A	N/A
	Kinar et al, 2017 [53]	Mixed	Maccabi Health Services EMRs, linked to the Israel Cancer Registry	133	112,451	N/A	N/A

^a^N/S: not stated.

^b^Cases of acute pancreatitis.

^c^NHS: National Health Service.

^d^Hospitals included: Northwest Prostate Institute Seattle, the University of Washington Seattle, the Johns Hopkins Hospital Baltimore, Memorial Sloan-Kettering Cancer Institute New York, Brigham and Women’s Hospital Boston, and The University of Texas MD Anderson Cancer Center

^e^NB: this study also included a small validation study in the Health Improvement Network database in the United Kingdom (n=25,613)

^f^EMR: electronic medical record.

^g^GP: general practitioner.

^h^N/A: not applicable

^i^EHR: electronic health record.

Almost all the studies used different data sets, with the exception of the Maccabi Health Services EHR, which was used in 2 studies [48,53]. The data set sizes ranged from 193 to 2,225,249 patients, with a mean of 241,585 (SD 555,953), median of 3,150, and IQR of 267,237 patients. The wide range is primarily due to the large data set used by Birks et al [52]. Of the 13 development studies, 3 provided no information on the control population used [39,46,51]. Five of the development studies did not provide full information on how they partitioned their data set for the training and testing of the algorithm [39,41,43,47,49]. Five studies appeared to have independent training and testing data sets, with most split in ratios ranging from 60:40 to 70:30 [40,44-46,50].

Three studies [34,52,53] validated a previously developed AI technique [48] in independent data sets. Kinar et al [48] reported both the initial development of an AI technique and a subsequent validation study in an independent data set. The study by Cooper et al [41] was the only study that developed an AI technique based on prospectively collected clinical data, with the data originating from a pilot study of fecal immunochemical testing by the NHS Bowel Cancer Screening Programme [116].

Table 4 summarizes the main reported outcome measures. Specificity (n=11), AUROC (n=11), and sensitivity (n=10) were the most frequently reported; others included PPV (n=6), NPV (n=5), diagnostic accuracy (n=4), and odds ratios (n=3). Specificity results range from 80.6% [45] to 100% [51], sensitivity results from 0% [51] to 96.7% [40], and AUROC results from 0.55 [45] to 0.9896 [44].

**Table 4:** Outcome measures.

Study		Cancer type	Outcome measures for each modality of AI^a^
**Development studies**			
	Alzubi et al, 2019 [39]	Lung cancer	Specificity: 92%, Accuracy: 93% False positive rate: 9%, F-1 score: 92%
	Chang et al, 2009 [40]	Pancreatic cancer	Sensitivity: BPNN^b^ 88.3%, genetic algorithm LR^c^ 96.7%, stepwise LR 96.7% Specificity: BPNN 84.2%, genetic algorithm LR 82.5%, stepwise LR 73.7% AUROC^d^: BPNN 0.895, genetic algorithm LR 0.921, stepwise LR 0.882
	Cooper et al, 2018 [41]	Colorectal cancer	Sensitivity: 35.15% (at FIT^e^ threshold 160 µg g^-1^) Specificity: 85.57% PPV^f^: 51.47%, NPV^g^: 75.19%, AUROC: 0.69, cancer detection rate: 10.66%
	Cowley et al, 2013 [42]	Colorectal cancer	Sensitivity: 90% Specificity: 96% PPV: 62%, NPV: 99%
	Daqqa et al, 2017 [43]	Leukemia	Sensitivity: SVM^h^ 69.7%, K-NN^i^ 60.0%, decision tree 62.4% Specificity: SVM 81.5%, K-NN 82.8%, decision tree 87.1% PPV: SVM 71.3%, K-NN 68.1%, decision tree 76.1% NPV: SVM 80.4%, K-NN 74.1%, decision tree 87.1% Accuracy: SVM 76.82%, K-NN 72.15%, decision tree 77.3% F-measure: SVM 70%, K-NN 60%, decision tree 67%
	Goryński et al, 2014 [44]	Lung cancer	AUROC: 0.9896
	Hart et al, 2018 [45]	Lung cancer	Sensitivity: ANN^j^ 75.30% Specificity: ANN 80.60% AUROC: ANN 0.86, RF^k^ 0.81, SVM 0.55
	Kalra et al, 2003 [46]	Prostate cancer	Specificity: 92% AUROC: 0.825
	Kang et al, 2017 [47]	Any cancer	Sensitivity: DNN^l^ 64.07%, SVM 54.46%, decision tree 60.00% Specificity: DNN 94.77%, SVM 95.27%, decision tree 91.50% AUROC: DNN 0.882, SVM 0.928, decision tree 0.824 Accuracy: DNN 86.00%, SVM 83.83%, decision tree 83.60% Using fuzzy interval of threshold with DNN achieves sensitivity 90.20%, specificity 94.22%, accuracy 93.22%
	Kinar et al, 2016 [48]	Colorectal cancer	Specificity: Testing set 88% overall (at a sensitivity of 50%). Higher for proximal colon tumors. Validation set 94% (at a sensitivity of 50%) AUROC: Testing set 0.82, validation set 0.81 OR^m^ 26 at false +ve rate of 0.5% (testing set), OR 40 at false +ve rate of 0.5% (validation set). Algorithm identified 48% more CRC^n^ cases than gFOBT^o^
	Kop et al, 2016 [49]	Colorectal cancer	Sensitivity: CART^p^ 53.9%, RF 63.7%, LR 64.2% PPV: CART 2.6%, RF 3%, LR 3% AUROC: CART 0.885, RF 0.889, LR 0.891 F1-score: CART 0.049, RF 0.057, LR 0.058. Drugs for constipation most important predictor of CRC, followed by iron deficiency anemia
	Miotto et al, 2016 [50]	Multiple diseases and cancers	Specificity: 92% AUROC: 0.773 for classification of all diseases (cancer and other diagnoses). Rectal or anal cancer 0.887, liver or intrahepatic bile duct cancer 0.886, prostate cancer 0.859, multiple myeloma 0.849, ovarian cancer 0.824, bladder cancer 0.818, testicular cancer 0.811, pancreatic cancer 0.795, leukemia 0.774, uterine cancer 0.771, non-Hodgkin lymphoma 0.771, bronchial or lung cancer 0.770, colon cancer 0.767, breast cancer 0.762, kidney or renal pelvis cancer 0.753, brain or nervous system cancer 0.742, Hodgkin disease 0.731, cervical cancer 0.675 Accuracy index: 0.929 overall for classification of all diseases F-score: 0.181 for classification of all diseases Deep patient obtained approximately 55% correct predictions when suggesting 3 or more diseases per patient, regardless of time interval
	Payandeh et al, 2009 [51]	CML^q^ and lymphopro-liferative disorders	Sensitivity: CML 0%, lymphoproliferative disorder 0% Specificity: CML 100%, lymphoproliferative disorder 99.2% PPV: CML 0%, lymphoproliferative disorder 0% NPV: CML 99.2%, lymphoproliferative disorder 100% Error % for convoluted neural network 0.33, error % for LR 0.78
**Validation studies**			
	Birks et al, 2017 [52]	Colorectal cancer	AUROC: analyzed at various time intervals before diagnosis, 3-6 months 0.844, 18-24 months 0.776
	Hornbrook et al, 2017 [34]	Colorectal cancer	Sensitivity: 0-180 days (test to diagnosis): 50-75 years: 34.5%, 40-89 years: 39.9%; 181-360 days: 50-75 years: 18.8%, 40-89 years: 27.4% AUROC: 0.80, OR: 34.7 at 99% specificity, 19.7 at 97%, 14.6 at 95%, 10.0 at 90%
	Kinar et al, 2017 [53]	Colorectal cancer	Sensitivity: 17.0% at 1% +ve rate, 24.4% at 3% +ve rate PPV: 2.1% at 1% +ve rate, 1.0% at 3% +ve rate NPV: 99.9% at 1% +ve rate, 99.9% at 3% +ve rate OR: 21.8% at 1% +ve rate, 10.9% at 3% +ve rate

^a^AI: artificial intelligence.

^b^BPNN: back propagation neural network.

^c^LR: logistic regression.

^d^AUROC: area under the receiver operating characteristic.

^e^FIT: fecal immunochemical test.

^f^PPV: positive predictive value.

^g^NPV: negative predictive value.

^h^SVM: support vector machine.

^i^K-NN: K-nearest neighbor.

^j^ANN: artificial neural network.

^k^RF: random forest.

^l^DNN: deep neural network.

^m^OR: odds ratio.

^n^CRC: colorectal cancer.

^o^gFOBT: guaiac fecal occult blood test.

^p^CART: classification and regression trees.

^q^CML: chronic myeloid leukemia.

We looked for other secondary outcomes, including implementation barriers to AI techniques in primary care settings, but did not find any evidence related to patient or clinician acceptability or cost-effectiveness.

Table 5 shows the outcomes of the risk of bias assessment using the QUADAS-2 tool. The studies demonstrated a wide range in quality; however, no studies were excluded based on their risk of bias assessment. The identified limitations were acknowledged in the relative contribution of the studies to the conclusions of the review.

**Table 5:** Critical appraisal results using the Quality Assessment of Diagnostic Accuracy Studies-2 tool.

Study	Risk of bias				Applicability concerns		
	Patient selection	Index test	Reference standard	Flow and timing	Patient selection	Index test	Reference standard
Alzubi et al, 2019 [39]	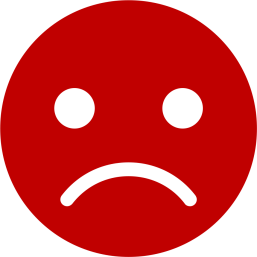 ^a^	 ^b^		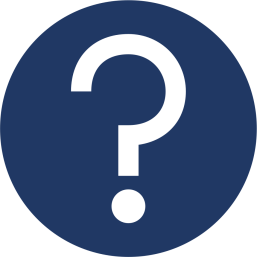 ^c^			
Birks et al, 2017 [52]							
Chang et al, 2009 [40]	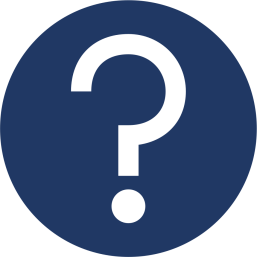		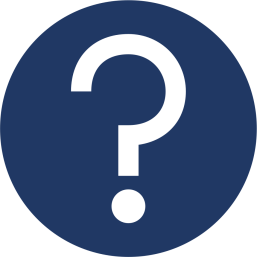	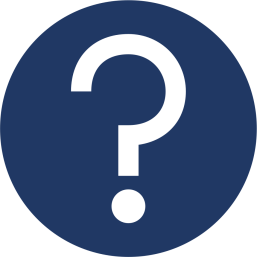			
Cooper et al, 2018 [41]							
Cowley et al, 2013 [42]							
Daqqa et al, 2017 [43]	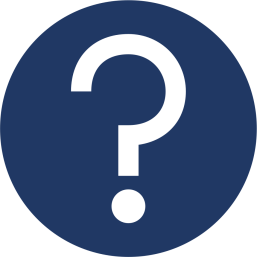		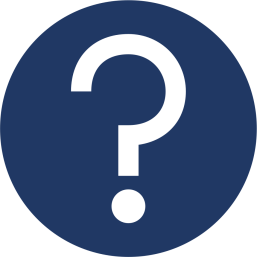	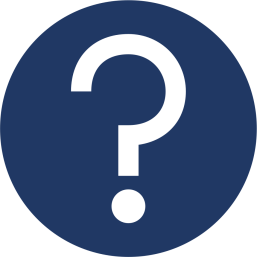			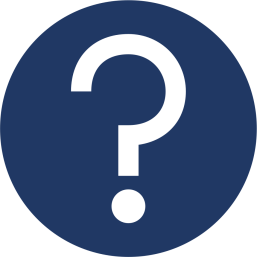
Goryński et al, 2014 [44]	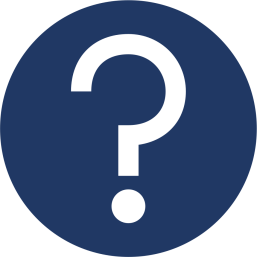		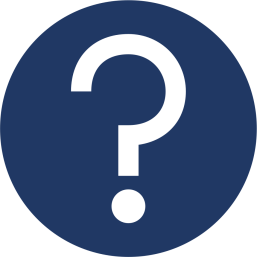				
Hart et al, 2018 [45]			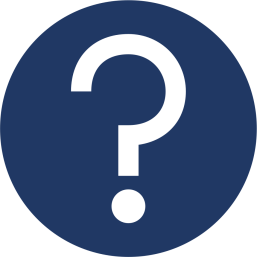				
Hornbrook et al, 2017 [34]							
Kalra et al, 2003 [46]							
Kang et al, 2017 [47]			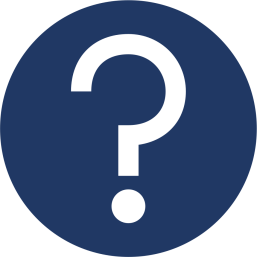	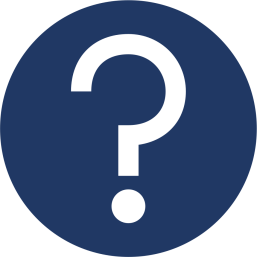			
Kinar et al, 2016 [48]							
Kinar et al, 2017 [53]							
Kop et al, 2016 [49]							
Miotto et al, 2016 [50]			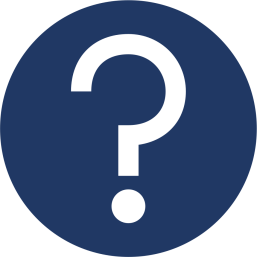				
Payandeh et al, 2009 [51]	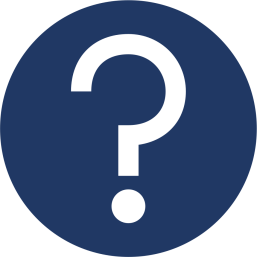		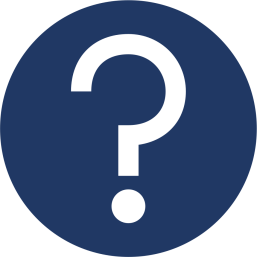				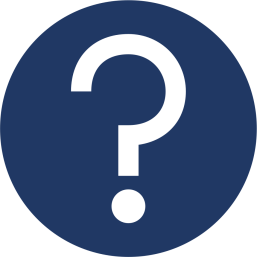

^a^High risk.

^b^Low risk.

^c^Unclear risk.

Table 6 summarizes the computer-based technologies identified in our parallel scoping review of commercial AI technologies. We identified 21 commercial computer-based technologies. Of these, 11 were clinician-facing differential diagnosis technologies that did not appear to be integrated into the EHR [117-127]. Ten of the technologies were linked to, or integrated into, the EHR in some way [8,128-136]. Nine of the technologies did not use AI algorithms incorporating an element of machine learning, as was required in our inclusion criteria [118,120-127]. It was also not clear from the websites and studies of 3 further technologies whether they met our AI inclusion criteria [117,130,134]. There were 8 technologies that met our inclusion criteria for AI (Abtrace [128], Babylon [8], Cthesigns [129], Isabel [131], Medial EarlySign [132], symcat [119], symptomate [135], and the unnamed technology evaluated by Liang et al [136]). Only the Medial EarlySign tool was evaluated for its performance in the diagnosis or triage of potential cancer [132]; 4 of the studies developing and validating this technology were included in this systematic review [34,48,52,53]. Cthesigns is specifically designed to aid the early diagnosis of cancer but has not been the subject of any studies we could identify [129].

**Table 6:** Summarizing scoping review of commercial artificial intelligence technologies.

Technology identified (origin) websites and associated academic studies		Not AI^a^		Not cancer		Not primary care based		Not early detection or diagnosis		Early research		Not published		Not primary research		<50 cases or controls	
**Abtrace (United Kingdom)**																	
	Abtrace website [128]		—^b^		—		—		—		—		X^c^		—		—
**Babylon (United Kingdom)**																	
	Babylon health website [8]		—		—		—		—		—		—		—		—
	Zhelezniak et al [137]		—		X		X		X		X		—		—		—
	Douglas et al [138]		—		X		X		X		X		—		—		—
	Smith et al [139]		—		X		X		X		X						
	National Health Service 111 powered by Babylon - Outcomes Evaluation [140]		—		X		—		X		—		—		—		—
	Middleton et al [141]		—		X		—		X		—		—		—		—
**Cthesigns (United Kingdom)**																	
	Cthesigns website [129]		—		—		—		—		—		X		—		—
**Diagnosis Pro (United States)**																	
	No website identified		—		—		—		—		—		—		—		—
	Bond et al [117]		N/C^d^		X		—		—		—		—		—		—
**DocResponse (United States)**																	
	Docresponse website [130]		N/C		—		—		—		—		X		—		—
**DxPlain (United States)**																	
	Dxplain website [118]		N/C		—		—		—		—		—		—		—
	Barnett et al [142]		X		—		—		—		X		—		X		—
	Barnett et al [143]		X		X		—		—		—		—		—		—
	Bauer et al [144]		X		X		—		—		—		—		—		—
	Berner et al [145]		X		X		X		—		—		—		—		—
	Bond et al [117]		X		X		—		—		—		—		—		X
	Elhanan et al [146]		X		—		—		—		X		—		—		—
	Elkin et al [147]		X		X		X		—		—		—		—		—
	Feldman et al [148]		X		X		X		—		—		—		—		X
	Hammersley et al [149]		X		X		X		—		—		—		—		—
	Hoffer et al [150]		X		—		—		X		—		—		—		—
	London et al [151]		X		—		—		—		X		—		—		—
**Iliad (United States)**																	
	No website identified		—		—		—		—		—		—		—		—
	Berner et al [145]		X		X		X		—		—		—		—		—
	Elstein et al [152]		X		X		X		—		—		—		—		X
	Friedman et al [153]		X		—		X		—		—		—		—		X
	Gozum et al [154]		X		—		X		—		—		—		—		X
	Graber et al [155]		X		—		X		—		—		—		—		X
	Heckerling et al [120]		X		—		X		—		—		—		—		X
	Lange et al [156]		X		—		—		—		—		—		—		X
	Lau et al [157]		—		—		—		—		—		—		X		—
	Li et al [158]		X		X		X		—		—		—		—		X
	Lincoln et al [159]		X		X		X		—		—		—		—		X
	Murphy et al [160]		X		X		X		—		—		—		—		X
	Wolf et al [161]		X		X		X		—		—		—		—		X
**Internist-1 (United States)**																	
	No website identified		—		—		—		—		—		—		—		—
	Miller et al [121]		X		X		X		—		—		—		—		X
	Miller et al [122]		X		—		X		—		—		—		—		X
**Isabel (United Kingdom)**																	
	Isabel healthcare website – Isabel pro [131]		—		—		—		—		—		—		—		—
	Bond et al [117]		—		X		—		—		—		—		—		—
	Ramnarayan et al [162]		—		X		—		—		—		—		—		—
	Ramnarayan et al [163]		—		X		—		—		—		—		—		—
	Carlson et al [164]		—		X		—		—		—		—		—		—
	Graber et al [165]		—		—		—		—		—		—		X		—
	Graber et al [166]		—		X		—		—		—		—		—		—
	Ramnarayan et al [167]		—		X		—		—		—		—		—		—
	Bavdekar et al [168]		—		X		—		—		—		—		—		—
	Ramnarayan et al [169]		—		X		—		—		—		—		—		—
	Semigran et al [20]		—		X		—		—		—		—		—		—
	Meyer et al [170]		—		X		—		—		—		—		—		—
**Meditel (United States)**																	
	No website identified		—		—		—		—		—		—		—		—
	Berner et al [145]		X		X		X		—		—		—		—		—
	Hammersley et al [149]		X		X		X		—		—		—		—		—
	Waxman et al [171]		X		X		X		—		—		—		—		—
	Wexler et al [123]		X		X		X		—		—		—		—		X
**Medial Early sign (United States/Israel)**																	
	Earlysign website [132]		—		—		—		—		—		—		—		—
	Kinar et al [53]^e^		—		—		—		—		—		—		—		—
	Birks et al [52]^e^		—		—		—		—		—		—		—		—
	Hornbrook et al [34]^e^		—		—		—		—		—		—		—		—
	Goshen et al [172]		—				X		—		—		—		—		—
	Zack et al [173]		—		X		—		—		—		—		—		—
	Cahn et al [174]		—		X		—		—		—		—		—		—
**Multilevel Diagnosis Decision Support System (Spain)**																	
	No website identified		—		—		—		—		—		—		—		—
	Rodriguez-Gonzalez et al [124]		X		X		—		—		—		—		—		X
**Online webGP (United Kingdom; later became eConsult)**																	
	Emis health online-triage website [175]^f^		—		—		—		—		—		—		—		—
	Hurleygroup website [176]^g^		—		—		—		—		—		—		—		—
	Edwards et al [133]		X		X		—		X		—		—		—		—
	Carter et al [177]		X		X		—		X		—		—		—		—
	Cowie et al [178]		X		X		—		X		—		—		—		—
**Pepid (United States)**																	
	Pepid website [125]^h^		N/C		—		—		—		—		—		—		—
	Bond et al [117]		X		X		X		—		—		—		—		—
**Problem Knowledge Couplers (PKC; United States)**																	
	No website identified		—		—		—		—		—		—		—		—
	Apkon et al [126]		X		—		—		X		—		—		—		—
**Quick Medical Reference (QMR) (United States; developed from Internist-1)**																	
	No website identified		—		—		—		—		—		—		—		—
	Arene et al [179]		X		—		X		—		—		—		—		X
	Bacchus et al [180]		X		—		X		—		—		—		—		X
	Bankowitz et al [181]		X		—		X		—		—		—		—		X
	Berner et al [145]		X		X		X		—		—		—		—		—
	Berner et al [182]		X		—		—		—		—		—		—		X
	Friedman et al [153]		X		—		X		—		—		—		—		X
	Gozum et al [154]		X		—		X		—		—		—		—		X
	Graber et al [155]		X		—		X		—		—		—		—		X
	Miller et al [122]		X		—		X		—		—		—		—		X
	Lemaire et al [183]		X		—		X		—		—		—		—		—
**Reconsider (United States)**																	
	No website identified		—		—		—		—		—		—		—		—
	Nelson et al [127]		X		X		X		—		—		—		—		—
**Symcat (United States)**																	
	Symcat website [119]		—		—		—		—		—		X		—		—
**Symptify (United States)**																	
	Symptify website [134]		N/C		—		—		—		—		X		—		—
**Symptomate (Poland)**																	
	Symptomate website [135]		—		—		—		—		—		X		—		—
**Unnamed**																	
	No website identified		—		—		—		—		—		—		—		—
	Liang H et al [136]		—		X		X		—		—		—		—		—

^a^AI: artificial intelligence.

^b^Not applicable or no data.

^c^Study excluded for the reason specified in the column label.

^d^N/C: not clear.

^e^These studies met the inclusion criteria of the systematic review and were therefore included.

^f^Edwards et al [133] suggests that this Egton Medical Information Systems (EMIS) application is powered by the eConsult system.

^g^Carter et al [177] suggests that this is the group who developed webGP.

^h^Several published studies are linked in the research section of the website, none involved use of the differential diagnosis or decision support tools. Some case studies audited the use of these tools.

## Discussion

### Principal Findings

We identified 16 studies reporting AI techniques that could facilitate the early detection of cancer and could be applied to the types of data found in primary care EHRs. However, heterogeneity of AI modalities, data set characteristics, outcome measures, conduct of these studies, and quality assessment meant that we were unable to draw strong conclusions about the utility of these techniques in primary care settings. There was a notable paucity of evidence on performance using primary care data. Coupled with the lack of evidence on implementation barriers or cost-effectiveness, this may help explain why AI techniques have not been adopted widely into primary care clinical practice to date. The study by Kinar et al [48] and its subsequent validation in independent data sets [34,52,53], including primary care data sets, is a valuable example of a staged evaluation of an AI technique from early development, via validation data sets, to evaluation in the population for intended use [22]. The work by Kop and collaborators [49,115,184] also represents a good example of the staged development of an AI technique, with sequential peer-reviewed, published evaluations at each stage.

We also identified 21 commercial AI technologies, many of which have not been evaluated and reported in peer-reviewed, published studies. Many other technologies that were patient-facing and designed for the triage of symptoms were identified but had not been applied to EHRs. Eight of these technologies appeared to be based on newer machine learning AI techniques, with the majority appearing to be driven by knowledge-based decision tree algorithms. Only one of the identified technologies has been evaluated specifically for cancer, although it may be more efficacious for these technologies to be very general in scope and to be widely used, rather than to have a narrow focus on cancer alone. With wider adoption, these technologies have a greater potential for raising patient and clinician awareness of cancer. However, it remains important to fully understand their diagnostic accuracy and safety, including for the triage of potential cancer symptoms. AI technologies applied to EHRs are potentially useful for primary care clinicians; however, they need to be designed in a way that is appropriate for the type and origin of the data found in primary care EHRs and to have been thoroughly and transparently evaluated in the population the technology is intended for.

### Strengths and Limitations

The strengths of this systematic review include the following: a broad and inclusive search strategy to avoid missing studies; guidance of an international expert panel in the development of the protocol and search strategy; independent screening, quality assessment, and data extraction processes; followed PRISMA guidance; and a parallel scoping review for commercial AI technologies. As only a few heterogeneous studies were identified, it was not possible to synthesize the data and evaluate the utility of these AI techniques. Furthermore, only one commercially available AI technology was identified via the systematic review. Many of the technologies identified in the parallel scoping review lacked sufficient academic detailing and evidence for their accuracy or safety. This is a rapidly evolving research area, which will require further review over time.

### Conclusions

Worldwide, there is a great deal of interest in AI techniques and their potential in medicine, not least in the United Kingdom where politicians and NHS leaders have publicly prioritized the incorporation of AI into clinical settings. Our findings support those of Kueper et al [17], namely, that although some AI techniques have good initial validation reports, they have not yet been through the steps for full application in clinical practice. Validation using independent data is preferable to splitting a single data set [185] and could be the next step in the development of many AI techniques identified in this review. Much of the research is at an early stage, with variable reporting and conduct, and requires further validation in prospective clinical settings and assessment of cost-effectiveness after clinical implementation before it can be incorporated into daily practice safely and effectively [186].

Consensus is required on how AI techniques designed for clinical use should be developed and validated to ensure their safety for patients and clinicians in their intended settings. Good internal and external validity is required in these experiments to avoid bias, most notably spectrum bias [187] and distributional shift [16], and to ensure that the appropriate data are used to develop the AI technique in keeping with its anticipated clinical setting and diagnostic function. The CanTest framework provides an outline for further studies aiming to develop this evidence base for AI techniques in clinical settings; to prove their safety and efficacy to commissioners, clinicians, and patients; and to enable them to be implemented in clinical practice [22]. Prospective evaluation in the clinical setting for which the AI technique is intended is essential: AI aimed at primary care clinics must be evaluated in primary care settings, where cancer prevalence is low compared with specialist settings, to accurately evaluate their future performance [187,188]. Further research around the acceptability of AI techniques for patients and clinicians and their cost-effectiveness will also be important to facilitate rapid implementation. Once these AI techniques are ready for implementation, they will require careful design to ensure effective integration into health information systems [189]. Data governance and protection must also be addressed, as they may present significant barriers to the implementation of these technologies [190,191].

In conclusion, AI techniques have the potential to aid the interpretation of patient-reported symptoms and clinical signs and to support clinical management, doctor-patient communication, and informed decision making. Ultimately, in the context of early cancer detection, these techniques may help reduce missed diagnostic opportunities and improve safety netting. However, although there are a few good examples of staged validation of these AI techniques, most of the research is at an early stage. We found numerous examples of the implementation of AI technologies without any or sufficient evidence for their accuracy or safety. Further research is required to build up the evidence base for AI techniques applied to EHRs and to reassure commissioners, clinicians, and patients that they are safe and effective enough to be incorporated into routine clinical practice.

## Appendix

Multimedia Appendix 1 Protocol for the study.

Multimedia Appendix 2 Search strategies.

Multimedia Appendix 3 Results of the full-text article review.

Multimedia Appendix 4 Supplementary information to table 1.

## Abbreviations

AI: artificial intelligence
AUROC: area under the receiver operating characteristic
CT: computed tomography
EHR: electronic health record
NHS: National Health Service
NIHR: National Institute for Health Research
NPV: negative predictive value
PPV: positive predictive value
PRISMA: Preferred Reporting Items for Systematic Reviews and Meta-analysis
QUADAS-2: quality assessment of diagnostic accuracy studies-2
