# Supplementary material for: Artificial Intelligence Techniques That May Be Applied to Primary Care Data to Facilitate Earlier Diagnosis of Cancer: Systematic Review
Source: J Med Internet Res. 2021 Mar 3;23(3):e23483. doi: 10.2196/23483 (PMC7970165; doi:10.2196/23483)
Supplement: Multimedia Appendix 1 [file jmir_v23i3e23483_app1.docx]

**Appendix 1 - Protocol for the study**

**Establishing which modalities of artificial intelligence (AI) for early detection and diagnosis of cancer are ready for implementation in primary care: a Systematic Review**

Owain T Jones, Charindu KI Ranmuthu, Kethaki Prathivadi, Smiji Saji, Natalia Calanzani, Jon Emery, Willie Hamilton, Hardeep Singh, Niek de Wit, Stephen Duffy, Fiona M Walter

**Background and Aims**

Approximately 360,000 people in the United Kingdom are diagnosed with cancer each year and approximately 160,000 people die of the disease. Half of the UK population over the age of 50 will be diagnosed with cancer in their lifetime.^1^ The NHS currently spends approximately £1 billion on cancer diagnostics per year;^2^ despite this, the UK lags behind comparable European nations with their cancer survival rates.^3^ Most people diagnosed with cancer in the UK first present in primary care,^4^ where General Practitioners (GPs) will evaluate (often vague) presenting symptoms and decide on an appropriate management strategy, including investigation/s, referral or reassurance. More accurate triage of these presenting symptoms could lead to earlier diagnosis of cancer, and improved outcomes for patients, including improved survival rates.^5,35^

The NHS long-term plan, published in January 2019, suggested that: *“Decision support and artificial intelligence are developing all the time. These technologies need to be embraced by the NHS... In the coming years AI (artificial technology) will make it possible for many tasks to be automated, quality to increase and staff to focus on the complexity of human interactions that technology will never master”*^36^

There is accumulating evidence that artificial intelligence (AI) can assist clinicians to make better clinical decisions or even replace human judgement in certain areas of healthcare. This is due to the increasing availability of healthcare data and rapid development of big data analytic methods. These AI technologies fall into several groups, including classical machine learning techniques, neural networks, and deep learning techniques: however, the rate of change of this research area means that new approaches are continuously being developed. There has been huge interest in the application of AI technologies, including machine learning and automated analysis approaches, to medical diagnosis. Recent studies have applied AI to medical records to improve the diagnosis of colorectal cancer;^34^ to patient symptoms to improve diagnosis; ^7,8^ and to images to improve diagnosis in diabetic retinopathy,^9^ mammography,^11^ computed tomography (CT) scans,^12^ acute lymphoblastic leukaemia,^13^ and skin cancer.^37,38^

There are currently no AI technologies that are established in routine clinical care. There are a variety of reasons for this, including some significant obstacles to implementation. These include: regulations which currently lack standards to assess the safety and efficacy of AI systems, uncertainty over the diagnostic accuracy and ethics of using AI systems in different healthcare and population settings; data sharing for further development and improvement of AI systems, and a lack of evidence about the costs and acceptability for implementation in the NHS.

Our aim is to focus on AI modalities for the early detection and diagnosis of cancer which are ready for possible implementation in primary care settings. We included AI-driven cancer risk prediction tools for the symptomatic population consulting in primary care, and AI-driven pattern recognition systems (e.g. applied to clinical or dermoscopic images to identify possible melanomas and other skin cancers in primary care).

**Methods**

We initially designed a search strategy for AI technologies for the detection of cancer in primary care in collaboration with University of Cambridge medical school librarians. Two researchers independently performed title and abstract screening of 2202 records to identify 128 articles for full text evaluation. However, many previously identified key research papers were not identified with this search strategy, and we concluded that the use of primary care terms in our search strategy was ineffective.

We presented this problem at the CanTest International School, an annual conference for cancer detection research in Primary Care that includes many senior researchers in this field. Peer feedback from this conference led us to re-evaluate the search strategy to focus on early detection instead of primary care. Feedback also led to a careful re-draft of the inclusion and exclusion criteria, to more fully define the forms of AI and the specific technology types we were interested in. This enabled us to exclude AI technologies types that were not feasible for implementation in primary care, for example, MRI and CT scans., and those not based on machine-learning approaches such as decision trees, rules-based systems and traditional epidemiological approaches to decision support. These inclusion criteria were carefully incorporated into the search strategy to refine the search, this was done by checking the first 100 articles after incorporation of the exclusion criteria to ensure at each stage that includable papers were not lost.

Full details of the search strategy are available in Appendix 2. The PICO framework for this search is:

P – Patients with symptoms which could indicate cancer

I – Artificial Intelligence based technologies suitable for implementation in primary care settings

C – Usual care

O – Early detection of cancer

We will search the following bibliographic databases from 1^st^ January 2000 to 11^th^ June 2019 for relevant published studies:

- Medline
- EMBASE
- SCOPUS
- Web of Science

**Inclusion and exclusion criteria**

The following inclusion criteria will be applied:

1. Primary research articles published in peer-reviewed journals, from 1^st^ January 2000 to 11^th^ June 2019,
2. Which provide evidence around the accuracy, utility, acceptability or cost-effectiveness of applying AI technologies to the early detection and diagnosis of cancer (as in Phase 2 and Phase 3 of the CanTest framework),
3. and these AI technologies may be considered for evaluation in primary care clinical settings, for example:
4. AI approaches applied to the Electronic Health Record (EHR) to improve cancer detection and diagnosis;
5. AI imaging technologies for detection of visible cancers (e.g. skin, oral, cervical cancers);
6. AI-based imaging technologies that are potentially implementable in primary care (e.g. ultrasound scans for thyroid cancer, breast cancer, sarcoma, intra-abdominal cancers etc.)

The following exclusion criteria will also be applied:

1. Studies that are not primary studies, including book chapters and unpublished conference abstracts;
2. Studies that only describe the development of an AI technology, without any testing or evaluation for effectiveness;
3. Studies describing the development of AI technologies that are not designed for, or not suitable for, the triage of symptomatic patients in primary care. For example, AI technologies that segment a region of interest in superficial or subsurface digital imaging, but do not classify the image as malignant/suspicious or benign;
4. Studies describing AI technologies that are not designed for, or not suitable for use in, the early detection of cancer in primary care. For example, for AI technologies based on histopathological images (including cytology); or based on images obtained from MRI, CT, mammography, microwave technologies, or contrast-US; and invasive procedures such as endoscopy, bronchoscopy, hysteroscopy, cystoscopy, trans-rectal US or trans-vaginal US;
5. Studies of technologies that do not include an element of ‘machine learning’. By this we mean there must be a process of training whereby the algorithm is able to modify the weighting of its own elements to better fit the data, without human involvement. This excludes rules-based systems and traditional epidemiological-based approaches.
6. Studies which use AI for biomarker discovery, including those used for blood, urine or exhaled breath clinical samples.
7. Studies based on sample sizes of less than 50 cases or less than 50 controls

The validity of the search strategy will be assessed by cross-checking articles identified in the search against the reference lists of key review articles. The search will then be manually extended by:

1. Hand-searching reference lists of included articles;
2. Hand-searching of key journals;
3. Hand-searching of existing networks (e.g. the International Skin Imaging Collaboration, Arxiv), organisations (e.g. Google, Microsoft, IBM etc) and conferences.

**Study Selection**

Study selection will proceed according to the following steps:

1. The titles and abstracts of the complete list of identified papers will be assessed by OTJ against the inclusion and exclusion criteria. We will conduct a verification exercise where 10% of the identified papers will be independently checked by two other researchers (SS, NMC). Those studies clearly not relevant will be rejected at this stage.
2. For studies where a definite decision to reject cannot be made based on title and abstract alone, the full paper will be obtained for detailed assessment.
3. One reviewer (OTJ, CKIR, KP, SS, NMC, FMW) will independently assess the full-text articles for the possibility of inclusion in the review, with at least 10% of the articles assessed by each researcher cross-checked by OTJ. Papers identified as not meeting the inclusion criteria will be excluded.
4. The researchers will meet regularly to discuss papers in which it is unclear whether they meet the inclusion criteria or not, any disagreements will be resolved by discussion and a consensus decision reached by the research team.

**Data Extraction**

The research team will determine which variables to extract in order to answer the research question and develop a data extraction spreadsheet (in Excel), piloted on 5 studies initially to determine whether the approach to data extraction is consistent with the research question and purpose.

The data extraction spreadsheet will facilitate systematic extraction of relevant information from papers identified as meeting the inclusion criteria. It will include details on:

1. Characteristics of the study
   1. Study details
   2. Year of Publication
   3. Location of the study
   4. Cancer looked at
   5. Intervention
      1. Modality of AI used
      2. Control test
   6. Study population
      1. Selection of participants (primary care, secondary care, images)
      2. Database used for images
      3. Disease positive population
      4. Disease negative population
      5. Training set
      6. Testing set
      7. Validation in a separate dataset
      8. Prospective evaluation in a clinical setting
2. Outcomes from the study
   1. Sensitivity
   2. Specificity
   3. PPV
   4. NPV
   5. AUROC
   6. Accuracy index
   7. Other
3. Challenges for implementation
   1. Data on acceptability of the technology to patients and/or clinicians or cost of the technology
   2. Other
4. Additional notes

Data extraction will be undertaken by two researchers independently (OTJ, CKIR, KP, SS, NMC, FMW). The research team will meet regularly to ensure consensus of views by discussing and resolving any differences in data extraction.

**Quality Assessment Framework**

Quality assessment of the included studies will be undertaken using the Quality Assessment of Diagnostic Accuracy Studies tool-2 (QUADAS-2, 2011). The aim of the quality assessment is to determine the validity of the results based on the design, methods, analysis, and conclusions of each study, and to assess the relative contribution of each study to the review. Limitations will be acknowledged but studies will not be excluded based on the quality assessment alone.

**Data Synthesis**

This stage will be refined depending on the data identified during the data extraction stage. From preliminary scoping searches we anticipate there will be few studies set in primary care populations, and that they will be heterogeneous in both study design and outcome measures. This will likely make meta-analysis unfeasible, in which case we will analyse the data using a narrative synthesis approach. We will aim to organise the literature thematically based on intervention types, giving an overview of the different interventions that are in development and how they inter-relate. We will also seek to summarize any numerical data found using tables and charts. These data will likely take the form of PPV, NPV, sensitivity, specificity, AUROC, but will depend on the data charted from the identified studies.
