# Supplementary material for: Artificial Intelligence Techniques That May Be Applied to Primary Care Data to Facilitate Earlier Diagnosis of Cancer: Systematic Review
Source: J Med Internet Res. 2021 Mar 3;23(3):e23483. doi: 10.2196/23483 (PMC7970165; doi:10.2196/23483)
Supplement: Multimedia Appendix 2 [file jmir_v23i3e23483_app2.docx]

**Appendix 2 – Search Strategies**

**Medline Search Strategy**

1. ((early or earlier or facilitat*or aid* or augment* or improv*) adj3 (detect* or diagnos* or recogni* or identif* or classif* or decision*)).mp. [mp=title, abstract, original title, name of substance word, subject heading word, floating sub-heading word, keyword heading word, organism supplementary concept word, protocol supplementary concept word, rare disease supplementary concept word, unique identifier, synonyms]
2. triage.mp. [mp=title, abstract, original title, name of substance word, subject heading word, floating sub-heading word, keyword heading word, organism supplementary concept word, protocol supplementary concept word, rare disease supplementary concept word, unique identifier, synonyms]
3. 1 or 2
4. cancer*.mp.
5. (tumour or tumor).mp.
6. neoplasm*.mp.
7. exp Neoplasms/
8. malignan*.mp.
9. exp Surgical Oncology/ or exp Medical Oncology/ or oncology.mp.
10. exp Carcinoma/ or carcinoma*.mp.
11. exp Sarcoma/ or sarcoma*.mp.
12. exp Melanoma/ or melanoma*.mp.
13. ((suspicious or pigmented) adj3 (skin tumour or skin lesion)).mp. [mp=title, abstract, original title, name of substance word, subject heading word, floating sub-heading word, keyword heading word, organism supplementary concept word, protocol supplementary concept word, rare disease supplementary concept word, unique identifier, synonyms]
14. leukaemia.mp. or exp Leukemia/
15. lymphoma.mp. or exp Lymphoma/
16. myeloma.mp
17. 4 or 6 or 7 or 8 or 9 or 10 or 11 or 12 or 13 or 14 or 15 or 16
18. artificial intelligence.mp. or exp Artificial Intelligence/
19. exp "Neural Networks (Computer)"/ or neural network*.mp.
20. machine learning.mp. or exp Machine Learning/
21. ((machine or computer) adj2 vision).mp. [mp=title, abstract, original title, name of substance word, subject heading word, floating sub-heading word, keyword heading word, organism supplementary concept word, protocol supplementary concept word, rare disease supplementary concept word, unique identifier, synonyms]
22. deep learning.mp. or exp Deep Learning/
23. exp Support Vector Machine/ or support vector machine*.mp.
24. 18 or 19 or 20 or 21 or 22 or 23
25. 3 and 17 and 24
26. limit 25 to yr="2000 -Current"

**Embase Search Strategy**

1. ((early or earlier or facilitat*or aid* or augment* or improv*) adj3 (detect* or diagnos* or recogni* or identif* or classif* or decision*)).ab,ti.
2. triage.ab,ti.
3. 1 or 2
4. cancer*.ab,ti.
5. (tumour or tumor).ab,ti.
6. "neoplasm*".ab,ti.
7. exp *neoplasm/
8. "malignan*".ab,ti.
9. oncology.ab,ti.
10. exp *oncology/
11. carcinoma.ab,ti.
12. exp *carcinoma/
13. sarcoma.ab,ti.
14. exp *sarcoma/
15. melanoma.ab,ti.
16. exp *melanoma/
17. ((suspicious or pigmented) adj3 (skin tumour or skin lesion)).ab,ti.
18. leukaemia.ab,ti.
19. exp *leukemia/
20. lymphoma.ab,ti.
21. exp *lymphoma/
22. myeloma.ab,ti.
23. 4 or 5 or 6 or 7 or 8 or 9 or 10 or 11 or 12 or 13 or 14 or 15 or 16 or 17 or 18 or 19 or 20 or 21 or 22
24. artificial intelligence.ab,ti.
25. exp *artificial intelligence/
26. "neural network*".ab,ti.
27. exp *artificial neural network/
28. machine learning.ab,ti.
29. exp *machine learning/
30. ((machine or computer) adj2 vision).ab,ti.
31. ((unsupervised or supervised or weak* supervised or limited supervision) adj3 (learning or network or algorithm)).ab,ti.
32. exp *computer assisted diagnosis/
33. deep learning.ab,ti.
34. support vector machines.ab,ti.
35. exp *support vector machine/
36. 24 or 25 or 26 or 27 or 28 or 29 or 30 or 31 or 32 or 33 or 34 or 35
37. 3 and 23 and 36
38. limit 37 to yr="2000 -Current"

**Web of Science Search Strategy**

1. TI=((early or earlier or facilitat or aid or augment or improv) NEAR/3 (detect or diagnos or recogni or identif or classif or decision))
2. TS=((early or earlier or facilitat or aid or augment or improv) NEAR/3 (detect or diagnos or recogni or identif or classif or decision))
3. TI=triage
4. TS=triage
5. #4 OR #3 OR #2 OR #1
6. TI=(cancer or tumor or tumour or neoplasm or malignancy or oncology or carcinoma or sarcoma or melanoma or leukaemia or leukemia or lymphoma or myeloma)
7. TS=(cancer or tumor or tumour or neoplasm or malignancy or oncology or carcinoma or sarcoma or melanoma or leukaemia or leukemia or lymphoma or myeloma)
8. TI=((suspicious or pigmented) NEAR/3 (tumour or tumor or lesion))
9. TS=((suspicious or pigmented) NEAR/3 (tumour or tumor or lesion))
10. #9 OR #8 OR #7 OR #6
11. TI=artificial intelligence OR TS=artificial intelligence
12. TI=neural network OR TS=neural network
13. TI=machine learning OR TS=machine learning
14. TI=((machine OR computer) NEAR/3 (vision)) OR TS=((machine OR computer) NEAR/3 (vision))
15. TI=((unsupervised or supervised or supervision) NEAR/3 (learning or network or algorithm)) OR TS=((unsupervised or supervised or supervision) NEAR/3 (learning or network or algorithm))
16. TI=(computer assisted diagnosis) OR TS=(computer assisted diagnosis)
17. TI=(deep learning) OR TS=(deep learning)
18. TI=(support vector machines) OR TS=(support vector machines)
19. #18 OR #17 OR #16 OR #15 OR #14 OR #13 OR #12 OR #11
20. #19 AND #10 AND #5

**Scopus Search Strategy**

( ( TITLE-ABS-KEY ( ( early OR earlier OR facilitat* OR aid* OR augment* OR improv* ) W/3 ( detect* OR diagnos* OR recogni* OR identif* OR classif* OR decision* ) ) AND PUBYEAR > 1999 ) OR ( TITLE-ABS-KEY ( triage ) AND PUBYEAR > 1999 ) )

AND

( ( TITLE-ABS-KEY ( ( cancer* OR tumour* OR tumor* OR neoplasm* OR malignan* OR oncology* OR carcinoma* OR sarcoma* OR melanoma* OR leukaemia OR leukemia OR lymphoma OR myeloma ) ) AND PUBYEAR > 1999 ) OR ( TITLE-ABS-KEY ( ( suspicious OR pigmented ) W/3 ( tumour OR tumor OR lesions ) ) AND PUBYEAR > 1999 ))

AND

( ( TITLE-ABS-KEY ( "artificial intelligence" ) AND PUBYEAR > 1999 ) OR ( TITLE-ABS-KEY ( "neural network*" ) AND PUBYEAR > 1999 ) OR ( TITLE-ABS-KEY ( "machine learning" ) AND PUBYEAR > 1999 ) OR ( TITLE-ABS-KEY ( ( machine OR computer ) W/2 vision ) AND PUBYEAR > 1999 ) OR ( TITLE-ABS-KEY ( ( unsupervised OR supervised OR supervision ) W/3 ( learning OR network OR algorithm ) ) AND PUBYEAR > 1999 ) OR ( TITLE-ABS-KEY ( "computer assisted diagnosis" ) AND PUBYEAR > 1999 ) OR ( TITLE-ABS-KEY ( "deep learning" ) AND PUBYEAR > 1999 ) OR ( TITLE-ABS-KEY ( "support vector machine*" ) AND PUBYEAR > 1999 ) )
