# Supplementary material for: Artificial Intelligence Techniques That May Be Applied to Primary Care Data to Facilitate Earlier Diagnosis of Cancer: Systematic Review
Source: J Med Internet Res. 2021 Mar 3;23(3):e23483. doi: 10.2196/23483 (PMC7970165; doi:10.2196/23483)
Supplement: Multimedia Appendix 3 [file jmir_v23i3e23483_app3.docx]

**Appendix 3 - Results of full text article review for all papers**

| **Paper** | **Include** | **Exclude** | **Reason** | Other comments |
| --- | --- | --- | --- | --- |
| Abdul Malik, N., Idris, W., Gunawan, T. S., Olanrewaju, R. F., & Noorjannah Ibrahim, S. (2018). Classification of normal and crackles respiratory sounds into healthy and lung cancer groups. International Journal of Electrical and Computer Engineering, 8(3), 1530–1538. https://doi.org/10.11591/ijece.v8i3.pp1530-1538 |  | 1 | <50 cases |  |
| Adams K, Sideris M, Papagrigoriadis S (2014) Can we make “Straight to Test” decisions in Two Week Wait (2WW) patients with the help of an Artificial Neural Network (ANN)? Colorectal Disease, 16(SUPPL. 2), 41. https://doi.org/http://dx.doi.org/10.1111/codi.12643 |  | 1 | Not published |  |
| Ahmed A, Shah MA, Wahid A, ul Islam S, Abbasi MK, Asghar MN (2017). Big data analytics using neural networks for earlier cancer detection. Journal of Medical Imaging and Health Informatics, 7(6), 1469–1474. https://doi.org/10.1166/jmihi.2017.2189 |  | 1 | Unable to access or obtain from British Library |  |
| Ahmed K, Al-Emran A, Jesmin T, Mukti RF, Rahman MZ, Ahmed F (2013). Early detection of lung cancer risk using data mining. Asian Pacific Journal of Cancer Prevention, 14(1), 595–598. https://doi.org/http://dx.doi.org/10.7314/APJCP.2013.14.1.595 |  | 1 | Not AI |  |
| Ahmed, U., Rasool, G., Zafar, S., Maqbool, H. F., & Ieee. (2019). Fuzzy Rule Based Diagnostic System to Detect the Lung Cancer. 2018 International Conference on Computing, Electronic and *Electrical Engineering*. Department of Mechatronics and Control Engineering, University of Engineering and Technology Lahore, Faisalabad Campus, Faisalabad, Pakistan: Institute of Electrical and Electronics Engineers Inc. https://doi.org/10.1109/ICECUBE.2018.8610976 |  | 1 | Not AI |  |
| Alaa, A. M., Moon, K. H., Hsu, W., & van der Schaar, M. (2016). ConfidentCare: A Clinical Decision Support System for Personalized Breast Cancer Screening. Ieee Transactions on Multimedia, 18(10), 1942–1955. https://doi.org/10.1109/tmm.2016.2589160 |  | 1 | Mammography required (not primary care) |  |
| Alharbi, A., Tchier, F., & Rashidi, M. (2016). Using a GeneticFuzzy Algorithm as a Computer Aided Breast Cancer Diagnostic Tool. Asian Pacific Journal of Cancer Prevention : APJCP, 17(7), 3651–3658. Retrieved from http://ovidsp.ovid.com/ovidweb.cgi?T=JS&PAGE=reference&D=med12&NEWS=N&AN=27510026 |  | 1 | Pathology |  |
| ALzubi, J. A., Bharathikannan, B., Tanwar, S., Manikandan, R., Khanna, A., & Thaventhiran, C. (2019). Boosted neural network ensemble classification for lung cancer disease diagnosis. Applied Soft Computing Journal, 80, 579–591. https://doi.org/10.1016/j.asoc.2019.04.031 | 1 |  |  |  |
| Ayeldeen, H., Elfattah, M. A., Shaker, O., Hassanien, A. E., & Kim, T.-H. (2016). Case-based retrieval approach of clinical breast cancer patients (pp. 38–41). Cairo University, Faculty of Computers and Information, Egypt: Institute of Electrical and Electronics Engineers Inc. https://doi.org/10.1109/CIA.2015.17 |  | 1 | Early research |  |
| Balachandran, K., & Anitha, R. (2013). An efficient optimization based lung cancer pre-diagnosis system with aid of feed forward back propagation neural network (FFBNN). Journal of Theoretical and Applied Information Technology, 56(2), 263–271. Retrieved from https://www.scopus.com/inward/record.uri?eid=2-s2.0-84886942617&partnerID=40&md5=0c14203eaf9e4f0952bd51864fff525d |  | 1 | <50 cases/controls |  |
| Bhat, J. A., George, V., Malik, B., & Ieee. (2015). Cloud computing with Machine Learning could help us in the early diagnosis of breast cancer. 2015 Second International Conference on Advances in Computing and *Communication Engineering Icacce 2015.* https://doi.org/10.1109/icacce.2015.62 |  | 1 | Pathology |  |
| Birks, J., Bankhead, C., Holt, T. A., Fuller, A., & Patnick, J. (2017). Evaluation of a prediction model for colorectal cancer: retrospective analysis of 2.5 million patient records. Cancer Medicine, 6(10), 2453–2460. https://doi.org/10.1002/cam4.1183 | 1 |  |  |  |
| Chang, C.-L., & Hsu, M.-Y. (2009). The study that applies artificial intelligence and logistic regression for assistance in differential diagnostic of pancreatic cancer. Expert Systems with Applications, 36(7), 10663–10672. https://doi.org/10.1016/j.eswa.2009.02.046 | 1 |  |  |  |
| Chauhan, R., Kaur, H., & Sharma, S. (2016). A feature based approach for medical databases. In K. M., G. V., & B. S.K. (Eds.) (Vol. 12-13-Augu). Dept of Biotechnology, Amity University, Noida, India: Association for Computing Machinery. https://doi.org/10.1145/2979779.2979873 |  | 1 | Not early detection |  |
| Chen, Y., & Joo, E. M. (2012). Biomedical diagnosis and prediction using parsimonious fuzzy neural networks (pp. 1477–1482). School of EEE, Nanyang Technological University, Singapore 639798, Singapore. https://doi.org/10.1109/IECON.2012.6388524 |  | 1 | Mammography required (not primary care) |  |
| Choudhury T, Kumar V, Nigam D, Mandal B. Intelligent Classification of Lung & Oral Cancer through Diverse Data Mining Algorithms," 2016 International Conference on Micro-Electronics and Telecommunication Engineering (ICMETE), Ghaziabad, 2016, pp. 133-138, doi: 10.1109/ICMETE.2016.24. |  | 1 | Unable to access |  |
| Çinar, M., Engin, M., Engin, E. Z., & Ziya Ateşçi, Y. (2009). Early prostate cancer diagnosis by using artificial neural networks and support vector machines. Expert Systems with Applications, 36(3 PART 2), 6357–6361. https://doi.org/10.1016/j.eswa.2008.08.010 |  | 1 | TRUS required (not primary care) |  |
| Cooper, J. A., Parsons, N., Stinton, C., Mathews, C., Smith, S., Halloran, S. P., … Taylor-Phillips, S. (2018). Risk-adjusted colorectal cancer screening using the FIT and routine screening data: development of a risk prediction model. British Journal of Cancer, 118(2), 285–293. https://doi.org/https://dx.doi.org/10.1038/bjc.2017.375 | 1 |  |  |  |
| Cowley J (2012) The use of knowledge discovery databases in the identification of patients with colorectal cancer (dissertation). University of Hull: https://doi.org/http://dx.doi.org/10.1111/codi.12260 | 1 |  |  |  |
| Daqqa, K. A. S. A., Maghari, A. Y. A., & Al Sarraj, W. F. M. (2017). Prediction and diagnosis of leukemia using classification algorithms (pp. 638–643). Faculty of Information Technology, Islamic University of Gaza, Gaza, Palestine: Institute of Electrical and Electronics Engineers Inc. https://doi.org/10.1109/ICITECH.2017.8079919 | 1 |  |  |  |
| Del Grossi, A. A., De Mattos Senefonte, H. C., & Quaglio, V. G. (2014). Prostate cancer biopsy recommendation through use of machine learning classification techniques. Lecture Notes in Computer Science (Including Subseries Lecture Notes in Artificial Intelligence and Lecture Notes in Bioinformatics). Universidade Estadual de Londrina, Rodovia Celso Garcia Cid, Km 380 - Campus Universitário, Londrina, 86057-970, Brazil: Springer Verlag. https://doi.org/10.1007/978-3-319-12027-0_57 |  | 1 | TRUS required (not primary care) |  |
| Durga, S., & Kasturi, K. (2017). Lung disease prediction system using data mining techniques. Journal of Advanced Research in Dynamical and Control Systems, 9(5), 62–66. Retrieved from https://www.scopus.com/inward/record.uri?eid=2-s2.0-85029003980&partnerID=40&md5=3479169265b3570a86e7f79420bb1f66 |  | 1 | Unable to access or obtain from British Library |  |
| Elhoseny, M., Bian, G.-B., Lakshmanaprabu, S. K., Shankar, K., Singh, A. K., & Wu, W. (2019). Effective features to classify ovarian cancer data in internet of medical things. Computer Networks, *159,* 147–156. https://doi.org/10.1016/j.comnet.2019.04.016 |  | 1 | Pathology |  |
| Elshazly, H. I., Elkorany, A. M., & Hassanien, A. E. (2013). Ensemble-based classifiers for prostate cancer diagnosis. In 2013 9th International Computer Engineering Conference: Today Information Society What’s Next?, *ICENCO 2013* (pp. 49–54). Faculty of Computers and Information, Cairo University, Cairo, Egypt: IEEE Computer Society. https://doi.org/10.1109/ICENCO.2013.6736475 |  | 1 | Pathology |  |
| Fan, Y.-J., & Chaovalitwongse, W. A. (2010). Optimizing feature selection to improve medical diagnosis. Annals of Operations Research, 174(1), 169–183. https://doi.org/10.1007/s10479-008-0506-z |  | 1 | Mammography required (not primary care) |  |
| Gaebel, J., Cypko, M. A., & Lemke, H. U. (2016). Accessing patient information for probabilistic patient models using existing standards. (A. E., S. G., H. A., & H. D., Eds.). Innovation Center Computer Assisted Surgery, University Leipzig, Semmelweisstraße 14, Leipzig, 04103, Germany: IOS Press. https://doi.org/10.3233/978-1-61499-645-3-107 |  | 1 | Early research |  |
| Gao, Z., Gong, J., Qin, Q., & Lin, J. (2005). [Application of support vector machine in the detection of early cancer]. Sheng Wu Yi Xue Gong Cheng Xue Za Zhi = Journal of Biomedical Engineering = Shengwu Yixue Gongchengxue *Zazhi*, *22*(5), 1045–1048. Retrieved from http://ovidsp.ovid.com/ovidweb.cgi?T=JS&PAGE=reference&D=med5&NEWS=N&AN=16294750 |  | 1 | Unable to access |  |
| Gao Z, Gong J, Qin Q, Lin J (2005). Application of support vector machine in the detection of early cancer. Sheng Wu Yi Xue Gong Cheng Xue Za Zhi = Journal of Biomedical Engineering = Shengwu Yixue Gongchengxue Zazhi, 22(5), 1045–1048. Retrieved from http://ovidsp.ovid.com/ovidweb.cgi?T=JS&PAGE=reference&D=emed9&NEWS=N&AN=41883151 |  | 1 |  | Duplicate of above paper |
| Gelnarová, E., & Šafařík, L. (2005). Comparison of three statistical classifiers on a prostate cancer data. Neural Network World, 15(4), 311–318. Retrieved from https://www.scopus.com/inward/record.uri?eid=2-s2.0-24644475153&partnerID=40&md5=a4810ddd6da74b281ef26d8b6277f205 |  | 1 | Not published |  |
| Ghaderzadeh, M. (2013). Clinical decision support system for early detection of prostate cancer from benign hyperplasia of prostate. In C. U. Lehmann, E. Ammenwerth, & C. Nohr (Eds.), Medinfo 2013: Proceedings of the 14th World Congress on Medical and Health Informatics, Pts 1 and 2 (Vol. 192, p. 928). Netherlands. https://doi.org/10.3233/978-1-61499-289-9-928 |  | 1 | Not published |  |
| Ghany, K. K. A., Ayeldeen, H., Zawbaa, H. M., Shaker, O., & Ieee. (2015). A rough set-based reasoner for medical diagnosis. In 2015 International Conference on Green Computing and Internet of Things (pp. 429–434). Faculty of Computers and Information, Beni-Suef University, Egypt: Institute of Electrical and Electronics Engineers Inc. https://doi.org/10.1109/ICGCIoT.2015.7380502 |  | 1 | Early research |  |
| Ghany, K. K. A., Ayeldeen, H., Zawbaa, H. M., Shaker, O., Ayedeen, G., Ieee, & Ayeldeen, G. (2017). Diagnosis of Breast Cancer using secured classifiers. In 2017 International Conference on Electrical and Computing Technologies and Applications (Vol. 2018-Janua, pp. 680–684). Faculty of Computers and Information, Beni-Suef University, Egypt: Institute of Electrical and Electronics Engineers Inc. https://doi.org/10.1109/ICECTA.2017.8251947 |  | 1 | Early research |  |
| Goraneseu, F., Gorunescu, M., El-Darzi, E., Ene, M., & Gorunescu, S. (2005). Statistical comparison of a probabilistic neural network approach in hepatic cancer diagnosis (Vol. I, pp. 237–240). Department of Mathematics, Biostatistics and Computer Science, University of Medicine and Pharmacy of Craiova, Romania. Retrieved from https://www.scopus.com/inward/record.uri?eid=2-s2.0-33847782793&partnerID=40&md5=dd5ea32a16ad07ff9bfb9f7099a37b56 |  | 1 | Not published |  |
| Gorunescu, F., & Belciug, S. (2016). Boosting backpropagation algorithm by stimulus-sampling: Application in computer-aided medical diagnosis. Journal of Biomedical Informatics, 63, 74–81. https://doi.org/10.1016/j.jbi.2016.08.004 |  | 1 | Mammography required (not primary care) |  |
| Gorunescu, M., Gorunescu, F., & Revett, K. (2006). A Neural Computing-Based Approach for the Early Detection of Hepatocellular Carcinoma. In C. Ardil (Ed.), Proceedings of World Academy of Science, Engineering and Technology, Vol 17 (Vol. 17, pp. 65-+). |  | 1 | <50 cases/controls |  |
| Goryński, K., Safian, I., Gra̧dzki, W., Marszałł, M. P., Krysiński, J., Goryński, S., … Romaszko, J. (2014). Artificial neural networks approach to early lung cancer detection. Central European Journal of Medicine, 9(5), 632–641. https://doi.org/http://dx.doi.org/10.2478/s11536-013-0327-6 | 1 |  |  |  |
| Govinda, K., Singla, K., & Jain, K. (2018). Fuzzy based uncertainty modeling of Cancer Diagnosis System (pp. 740–743). SCOPE, VIT, Vellore, India: Institute of Electrical and Electronics Engineers Inc. https://doi.org/10.1109/ISS1.2017.8389272 |  | 1 | Pathology |  |
| Halpern, Y., Horng, S., Choi, Y., & Sontag, D. (2016). Electronic medical record phenotyping using the anchor and learn framework. Journal of the American Medical Informatics Association, 23(4), 731–740. https://doi.org/10.1093/jamia/ocw011 |  | 1 | Not early detection |  |
| Hart, G. R., Roffman, D. A., Decker, R., & Deng, J. (2018). A multi-parameterized artificial neural network for lung cancer risk prediction. PloS One, 13(10), e0205264. https://doi.org/https://dx.doi.org/10.1371/journal.pone.0205264 | 1 |  |  |  |
| Hart GR, Roffman DA, Decker R (2018) Stratification of lung cancer risk from personal health data. Medical Physics, 45(6), e446–e447. https://doi.org/http://dx.doi.org/10.1002/mp.12938 |  | 1 | Not published |  |
| Hornbrook, MC, Goshen R, Choman E, O'Keeffe-Rosetti, Kinar Y, Liles EG, Rust KC (2017). Early Colorectal Cancer Detected by Machine Learning Model Using Gender , Age , and Complete Blood Count Data. Digestive Diseases and Sciences, 62(10), 2719–2727. https://doi.org/10.1007/s10620-017-4722-8 | 1 |  |  |  |
| Hornbrook, MC, Goshen R, Choman E, O'Keeffe-Rosetti, Kinar Y, Liles EG, Rust KC (2018) Early Colorectal Cancer Detected by Machine Learning Model Using Gender, Age, and Complete Blood Count Data. Digestive Diseases and Sciences, 63(1), 2719–2727. https://doi.org/10.1007/s10620-017-4859-5 |  | 1 | Not primary research (correction to above article) |  |
| Hsu, J.-L., Hung, P.-C., Lin, H.-Y., & Hsieh, C.-H. (2015). Applying under-sampling techniques and cost-sensitive learning methods on risk assessment of breast cancer. Journal of Medical Systems, 39(4), 210. https://doi.org/https://dx.doi.org/10.1007/s10916-015-0210-x |  | 1 | Early research |  |
| Ilhan, H. O., & Celik, E. (2017). The mesothelioma disease diagnosis with artificial intelligence methods. Department of Computer Engineering, Yildiz Technical University, Istanbul, Turkey: Institute of Electrical and Electronics Engineers Inc. https://doi.org/10.1109/ICAICT.2016.7991825 |  | 1 | CT required (not primary care) |  |
| Ji, Z., & Wang, B. (2014). Identifying potential clinical syndromes of hepatocellular carcinoma using PSO-based hierarchical feature selection algorithm. BioMed Research International, 2014, 127572. https://doi.org/https://dx.doi.org/10.1155/2014/127572 |  | 1 | CT required (not primary care) |  |
| Kalra, P., Togami, J., Bansal B S, G., Partin, A. W., Brawer, M. K., Babaian, R. J., … L.S., R. (2003). A Neurocomputational Model for Prostate Carcinoma Detection. Cancer, 98(9), 1849–1854. https://doi.org/http://dx.doi.org/10.1002/cncr.11748 | 1 |  |  |  |
| Kang, G., & Ni, Z. (2017). Research on early risk predictive model and discriminative feature selection of cancer based on real-world routine physical examination data. In B. K., Z. Q., L. Y., T. T., W. Y., H. X.T., … W. G. (Eds.) (pp. 1512–1519). Key Laboratory of Universal Wireless Communications, Ministry of Education, Beijing University of Posts and Telecommunications, Beijing, China: Institute of Electrical and Electronics Engineers Inc. https://doi.org/10.1109/BIBM.2016.7822746 | 1 |  |  |  |
| Kinar, Y., Akiva, P., Choman, E., Kariv, R., Shalev, V., Levin, B., … Goshen, R. (2017). Performance analysis of a machine learning flagging system used to identify a group of individuals at a high risk for colorectal cancer. PloS One, 12(2), e0171759. https://doi.org/https://dx.doi.org/10.1371/journal.pone.0171759 | 1 |  |  |  |
| Kinar, Y., Kalkstein, N., Akiva, P., Levin, B., Half, E. E., Goldshtein, I., … Shalev, V. (2016). Development and validation of a predictive model for detection of colorectal cancer in primary care by analysis of complete blood counts: a binational retrospective study. Journal of the American Medical Informatics Association : JAMIA, 23(5), 879–890. https://doi.org/https://dx.doi.org/10.1093/jamia/ocv195 | 1 |  |  |  |
| Kong, Q., Wang, D., Wang, Y., Jin, Y., & Jiang, B. (2018). Multi-objective neural network-based diagnostic model of prostatic cancer. Xitong Gongcheng Lilun yu Shijian/System Engineering Theory and Practice, 38(2), 532–544. https://doi.org/10.12011/1000-6788(2018)02-0532-13 |  | 1 | Not early detection |  |
| Kop, R., Hoogendoorn, M., Teije, A. Ten, Buchner, F. L., Slottje, P., Moons, L. M. G., & Numans, M. E. (2016). Predictive modeling of colorectal cancer using a dedicated pre-processing pipeline on routine electronic medical records. Computers in Biology and Medicine, *76,* 30–38. https://doi.org/https://dx.doi.org/10.1016/j.compbiomed.2016.06.019 | 1 |  |  |  |
| Kou, L., Yuan, Y., Sun, J., & Lin, Y. (2018). Prediction of cancer based on mobile cloud computing and SVM (Vol. 2018-Janua, *pp*. 73–76). School of Computer Science and Technology, Harbin Engineering University, Hei Longjiang, Harbin, China: Institute of Electrical and Electronics Engineers Inc. https://doi.org/10.1109/DSA.2017.20 |  | 1 | Pathology |  |
| Kshivets O (2018) Precise Early Detection of Lung Cancer and Blood Cell Circuit. Journal of Thoracic Oncology, 13(10 Supplement), S783. https://doi.org/http://dx.doi.org/10.1016/j.jtho.2018.08.1360 |  | 1 | Not published |  |
| Liu, S., Gaudiot, J.-L., & Cristini, V. (2006). Prototyping Virtual Cancer Therapist (VCT): A software engineering approach (pp. 5424–5427). Henry Samueli School of Engineering, U.C. Irvine, United States. https://doi.org/10.1109/IEMBS.2006.259230 |  | 1 | Not early detection |  |
| Liu, Y., Pan, Q., & Zhou, Z. (2018). Improved feature selection algorithm for prognosis prediction of primary liver cancer. (P. C., H. T., & S. Z., Eds.). School of Computer Science and Information Engineering, Shanghai Institute of Technology, Shanghai, 201418, China: Springer New York LLC. https://doi.org/10.1007/978-3-030-01313-4_45 |  | 1 | Not AI/early research |  |
| Meng, J., Zhang, R., & Chen, D. (2019). Utilizing Narrative Text from Electronic Health Records for Early Warning Model of Chronic Disease (pp. 4210–4215). School *of* Economics and Management, Beijing Jiaotong University, Beijing, China: Institute of Electrical and Electronics Engineers Inc. https://doi.org/10.1109/SMC.2018.00713 |  | 1 | Not early detection/not primary care |  |
| Mesrabadi, H. A., & Faez, K. (2019). Improving early prostate cancer diagnosis by using Artificial Neural Networks and Deep Learning. In T. H. (*Ed*.) (pp. 39–42). Faculty of Computer and Information Technology, Islamic Azad University, Qazvin, Iran: Institute of Electrical and Electronics Engineers Inc. https://doi.org/10.1109/ICSPIS.2018.8700542 |  | 1 | Early research/<50cases/controls |  |
| Miotto R, Li L, Kidd BA, Dudley JT (2016) Deep Patient: An Unsupervised Representation to Predict the Future of Patients from the Electronic Health Records. Scientific Reports, 6, 26094. https://doi.org/10.1038/srep26094 | 1 |  |  |  |
| Morgado, P., Vicente, H., Abelha, A., Machado, J., Neves, J., & Neves, J. (2017). A case-based approach to colorectal cancer detection. (K. K. & J. N., Eds.). Centro Algoritmi, Universidade do Minho, Braga, Portugal: Springer Verlag. https://doi.org/10.1007/978-981-10-4154-9_50 |  | 1 | Pathology |  |
| Nalluri, M. R., Kannan, K., Manisha, M., & Roy, D. S. (2017). Hybrid Disease Diagnosis Using Multiobjective Optimization with Evolutionary Parameter Optimization. Journal of *Healthcare Engineering*, *2017.* https://doi.org/10.1155/2017/5907264 |  | 1 | Not early detection (various databases but either not cancer/not primary care/not early detection) |  |
| Nikitaev, V. G., Pronichev, A. N., Nagornov, O. V, Zaytsev, S. M., Polyakov, E. V, Romanov, N. A., … Levadnaya, M. G. (2019). Decision support system in urologic cancer diagnosis (Vol. 1189). National Research Nuclear University, MEPhI (Moscow Engineering Physics Institute), Kashirskoe shosse 31, Moscow, 115409, Russian Federation: Institute of Physics Publishing. https://doi.org/10.1088/1742-6596/1189/1/012032 |  | 1 | Pathology |  |
| Payandeh M, Aeinfar M, Aeinfar V, Hayati M (2010) A new method for diagnosis and predicting blood disorder and cancer using artificial neural networks based on H1 blood test results. International Journal of Laboratory Hematology, 32(SUPPL. 1), 157. https://doi.org/http://dx.doi.org/10.1111/j.1751-553X.2010.01241.x | 1 |  |  |  |
| Polat, K., & Senturk, U. (2018). A Novel ML Approach to Prediction of Breast Cancer: Combining of mad normalization, KMC based *feature* weighting and AdaBoostM1 classifier. Deparment of Electrical and Electronics Engineering, *Bolu* Abant Izzet Baysal University, Bolu, Turkey: Institute of Electrical and Electronics Engineers Inc. https://doi.org/10.1109/ISMSIT.2018.8567245 |  | 1 | Biomarkers |  |
| Rahman, M. A., & Muniyandi, R. C. (2018). Feature selection from colon cancer dataset for cancer classification using Artificial Neural Network. International Journal on Advanced Science, Engineering and Information Technology, *8*(4–2), 1387–1393. Retrieved from https://www.scopus.com/inward/record.uri?eid=2-s2.0-85055347743&partnerID=40&md5=616f738dbdff7bace28423d887ec8933 |  | 1 | Pathology |  |
| Ramya Devi, M., & Gomathy, B. (2015). An intelligent system for the detection of breast cancer using feature selection and PCA methods. International Journal of Applied Engineering Research, 10(20), 16874–16876. Retrieved from https://www.scopus.com/inward/record.uri?eid=2-s2.0-84942847127&partnerID=40&md5=26e6ce27fb63932bd79ddbf241c47a56 |  | 1 | Mammography required (not primary care) |  |
| Richter AN, Khoshgoftaar TM (2019) Melanoma risk modeling from limited positive samples. Network Modeling Analysis in Health Informatics and Bioinformatics, 8(1), 7. https://doi.org/http://dx.doi.org/10.1007/s13721-019-0186-4 |  | 1 | Pathology |  |
| Safdari R, Arpanahi HK, Langarizadeh M, Ghazisaiedi M, Dargahi H, Zendehdel K (2018) Design a fuzzy rule-based expert system to aid earlier diagnosis of gastric cancer. Acta Informatica Medica, 26(1), 19–23. https://doi.org/http://dx.doi.org/10.5455/aim.2018.26.19-23 |  | 1 | Not AI/<50cases & controls |  |
| Shalev V, Kinar Y, Kalkstein N, Akiva P, Half E, Goldshtein I, Chodick G. (2013) Computational analysis of blood counts significantly increases detection rate of gastric and colorectal cancers: PR0195 Esophageal, Gastric and Duodenal Disorders. Journal of Gastroenterology and Hepatology. 2013 Oct;28:761-2. |  | 1 | Not published |  |
| Sobar, Machmud, R., & Wijaya, A. (2016). Behavior determinant based cervical cancer early detection with machine learning algorithm. Advanced Science Letters, 22(10), 3120–3123. https://doi.org/10.1166/asl.2016.7980 |  | 1 | <50 cases |  |
| Soliman, T. H. A., Mohamed, R., & Sewissy, A. A. (2017). A hybrid Analytical Hierarchical Process and Deep Neural Networks approach for classifying breast cancer. In Z. A.M., E.-K. M.W., A. H.M., B. E.-D. A.M., & T. M. (Eds.) (pp. 212–219). Information Systems Dept., Faculty of Computers and Information, Assiut University, Egypt: Institute of Electrical and Electronics Engineers Inc. https://doi.org/10.1109/ICCES.2016.7822002 |  | 1 | Pathology |  |
| Sushma Rani, N., Srinivasa Rao, P., Parimala, P., Rani, S. N., Rao, P. S., & Parimala, P. (2017). An Efficient Statistical Computation Technique for Health Care Big Data Using R. In M. M. Noor, V. N. Mani, M. S. Ganesh, & M. H. Idris (Eds.), *International Conference on Materials, Alloys and Experimental Mechanics* (Vol. 225, p. 012159). https://doi.org/10.1088/1757-899x/225/1/012159 |  | 1 | Pathology |  |
| Wang, D., Quek, C., See Ng, G., & Geok See, N. (2014). Ovarian cancer diagnosis using a hybrid intelligent system with simple yet convincing rules. Applied Soft Computing, 20, 25–39. https://doi.org/10.1016/j.asoc.2013.12.018 |  | 1 | Biomarkers |  |
| Wang, G., Teoh, J. Y.-C., & Choi, K.-S. (2018). Diagnosis of prostate cancer in a Chinese population by using machine learning methods. Conference Proceedings : ... Annual International Conference of the IEEE Engineering in Medicine and Biology Society. *IEEE Engineering in Medicine and Biology Society. Annual Conference*, *2018,* 1–4. https://doi.org/https://dx.doi.org/10.1109/EMBC.2018.8513365 |  | 1 | TRUS required (not primary care) |  |
| Xu W, Zhang R, Qimin E, Liu J, Laing C (2015) The clinical application of data mining in laryngeal cancer. Lin Chuang Er Bi Yan Hou Tou Jing Wai Ke Za Zhi = Journal of Clinical Otorhinolaryngology, Head, and Neck Surgery, 29(14), 1272–1275. Retrieved from http://ovidsp.ovid.com/ovidweb.cgi?T=JS&PAGE=reference&D=emed16&NEWS=N&AN=610828357 |  | 1 | Not AI/not primary care |  |
| Yasodha, P., & Ananthanarayanan, N. R. (2015). Analysing big data to build knowledge based system for early detection of ovarian cancer. Indian Journal of Science and Technology, 8(14). https://doi.org/10.17485/ijst/2015/v8i14/65745 |  | 1 | Pathology |  |
| Zangooei, M. H., Habibi, J., & Alizadehsani, R. (2014). Disease Diagnosis with a hybrid method SVR using NSGA-II. Neurocomputing, 136, 14–29. https://doi.org/10.1016/j.neucom.2014.01.042 |  | 1 | Pathology |  |
| Zhang, L., Wang, H., Liang, J., & Wang, J. (2008). Decision Support in Cancer Base on Fuzzy Adaptive PSO for Feedforward Neural Network Training. (F. Yu, W. Chen, Z. Chen, & J. Yuan, Eds.), *Iscsct 2008: International Symposium on Computer Science and Computational Technology, Vol 1, Proceedings*. IEEE. https://doi.org/10.1109/iscsct.2008.73 |  | 1 | Pathology |  |
| Zhang, Z., Zhang, H., & Bast Jr., R. C. (2000). Application of *artificial* neural networks in ovarian cancer early detection (Vol. 4, pp. 107–112). Medical Univ of South Carolina, Charleston, *United* States: IEEE. Retrieved from https://www.scopus.com/inward/record.uri?eid=2-s2.0-0033686670&partnerID=40&md5=88015d1da572eea82e1b70481ac72087 |  | 1 | Not published |  |
