# Supplementary material for: Artificial Intelligence Techniques That May Be Applied to Primary Care Data to Facilitate Earlier Diagnosis of Cancer: Systematic Review
Source: J Med Internet Res. 2021 Mar 3;23(3):e23483. doi: 10.2196/23483 (PMC7970165; doi:10.2196/23483)
Supplement: Multimedia Appendix 4 [file jmir_v23i3e23483_app4.docx]

**Appendix 4 – Supplementary information**

| Supplementary information on the variables included in the AI techniques to complement table 1 | | | | | | | | | | | | | | | | | | | |  |
| --- | --- | --- | --- | --- | --- | --- | --- | --- | --- | --- | --- | --- | --- | --- | --- | --- | --- | --- | --- | --- |
| Authors | Comparison/control | | | | | | | | | Variables included | | | | | | | | | | |
|  | Histopathology | | | Specialist | | Clinical control not stated | | Other | | Age | Sex | Demographics | Symptoms | Co-morbidities | Lifestyle | Examination | FBC | Other blood tests | Other |  |
| Alzubi *et al.* 2019[39] | | x |  | |  | | 1 | | x | | … | … | Pain, haemoptysis, dyspnoea, cough, weakness | Asthma, Peripheral arterial disease | Smoking | … | … | … | FVC |  |
| Chang *et al.* 2009[40] | |  |  | | x | | 2,3 | | x | | X | … | Dull abdomen, severe vomiting, radiation of pain, aggravating/relieving factors, change in bowel habit, weight loss, palpable mass, fullness, anorexia, epigastric pain, fever, nausea, chills, LUQ pain, cold sweats, diarrhoea, tea coloured urine, yellow skin, fainting, vague symptoms, ascites, dyspepsia, jaundice, dyspnoea, blood in stool, tarry stool, poor appetite, RUQ pain, clay-colour stool, grey coloured stool, steatorrhoea | Hypertension, diabetes, mitral valve prolapse, hypercholesterolaemia, gall bladder stones, chronic Hepatitis C, chronic Hepatitis B, alcoholism, CVA | Smoking, alcohol | … | WBCs | Albumin, ALP, ALT, amylase, AST, Bilirubin (D- and T-), GGT, lipase | … |  |
| Cooper *et al.* 2018[41] | | x | x | |  | | 4 | | x | | X | IMD, previous screening history | … | … | … | … | … | … | FIT result |  |
| Cowley *et al.* 2013[42] | |  | x | |  | | 2,5 | | … | | … | … | PR bleeding, Mucus PR, change in bowel habit, straining or pain on defecation, tenesmus, incontinence, abdominal pain, tiredness, SOB, weight loss, decreased appetite | Personal or family history of polyps/bowel Cancer/other Cancer/IBD | Smoking | … | … | … | Aspirin use, analgesic use |  |
| Daqqa *et al.* 2017[43] | | x |  | |  | | 2 | | … | | … | … | … | … | … | … | x | … | … |  |
| Goryński *et al.* 2014[44] | | x | x | |  | |  | | x | | X | Education, place of residence, | Cough, dyspnoea, haemoptysis, weight loss, lack of appetite, chest pain, fever, bone pain, weakness, hoarseness >1 month, Zubrod score (performance status) | COPD, previous pulmonary TB, other lung condition, history of cancer in other organ, family hx of lung cancer, pneumonia, cardiovascular disease | Smoking, occupational exposure | Height, Weight, auscultatory changes, supraclavicular swollen glands, central nervous system signs | Hb, RBCs, Hct, WBCs, platelets | ESR, creatinine, Glutamic Oxoloacetic Transaminase, Na, K | Spirometry: FEV1, FEV1/FVC, FVC, FVC%, VC, VC%. X-ray changes |  |
| Hart *et al.* 2018[45] | |  |  | | x | | 2,6 | | x | | X | Ethnicity | … | Diabetes, emphysema, asthma, hypertension, heart disease, history of stroke | Smoking, exercise habits | BMI | … | … | … |  |
| Kalra *et al.* 2003[46] | | x |  | |  | | 2,3 | | x | | … | Ethnicity | International Prostate Symptom Score (IPSS) | Family history of prostate cancer | … | Digital Rectal Examination | … | Total PSA, Complexed PSA | … |  |
| Kang *et al.* 2017[47] | | x | x | |  | | 2 | | x | | X | … | … | … | … | BMI, Systolic BP, Diastolic BP | Hb, Hct, MCV, MCH, MCHC, RDW-CV, RDW-SD, WBC, GØ, LØ, MØ, platelets, MPV, PDW | Urea, Creatinine, Uric Acid, Fasting Blood Glucose, ALT, Triglycerides, Total Cholesterol. AFP, CEA | Urine dipstick: pH and specific gravity (acidity and conc.) |  |
| Kinar *et al.* 2016[48] | | x | x | |  | | 3,6 | | x | | X | … | … | … | … | … | Hb, RBC, Hct, MCV, MCH, MCHC, RDW, WBC, EØ, NØ, MØ, BØ, LØ, platelets, MPV | … | … |  |
| Kop *et al.* 2016[49] | | x | x | |  | |  | | x | | X | … | x | x | x | x | x | x | Medication, referrals |  |
| Miotto *et al.* 2016[50] | |  | x | |  | | 2,3 | | … | | … | x | x | x | x | x | … | x | Medication history |  |
| Payandeh *et al.* 2009[51] | | x | x | |  | | 3 | | … | | … | … | … | … | … | … | Hb, RBC, MCV, MCH, MCHC, RDW, WBC, NØ, LØ, leukØ, platelets | … | … |  |
| Birks *et al.* 2017[52] | | x | x | |  | |  | | X  YoB | | X | … | … | … | … | … | x | … | … |  |
| Hornbrook *et al.* 2017[34] | | x | x | |  | |  | | X  YoB | | X | … | … | … | … | … | x | … | … |  |
| Kinar *et al.* 2017[53] | | x | X | |  | |  | | x | | X | … | … | … | … | … | x | … | … |  |
| ^Modality of AI: WONN-MLB=Weight Optimized Neural Network with Maximum Likelihood Boosting, BPNN=Back Propogation Neural Network, LR=Logistic Regression, ANN=Artificial Neural Network, CVT=Cross Validation Techniques, BPANN=Back Propogation Artificial Neural Network, SVM=Support Vector Machine, DT=Decision Tree, KNN=K-Nearest Neighbour, MLPANN= Multi-Layer Perceptron Artificial Neural Network, RF=Random Forest, GBM=Gradient Boosting Model, CART=Classification And Regression Trees, DNN=Deep Neural Network^  ^Comparison/control ‘other’ key: 1=previously developed AI methods, 2=other AI methods developed by this author, 3=other statistical (i.e. non-AI) techniques, 4=colonoscopy, 5=primary care clinicians, 6=Screening tests (e.g. low Dose CT scan, Faecal Occult Blood Test)^  ^Key:^  ^FBC=Full Blood Count, Hb=Haemoglobin, RBC=Red Blood Cell Count, Hct=Haematocrit, MCV=Mean Corpuscular Volume, MCH=Mean Corpuscular Haemoglobin, MCHC=Mean Corpuscular Haemoglobin Concentration, RDW=Red Cell Distribution Width, RDW-CV=Red Cell Distribution Width – Co-efficient of Variation, RDW-SD= Red Cell Distribution Width – Standard Deviation, WBC=White Blood Cell Count, GØ=Granulocytes, LØ=Lymphocytes, MØ=Monocytes, EØ=Eosinophils, NØ=Neutrophils, BØ=Basophils, leukØ=Leukocytes, Platelets=Platelet Count, MPV=Mean Platelet Volume, PDW=Platelet Distribution Width,^  ^AFP=Alpha Fetoprotein, AI=Artificial intelligence, ALP=Alkaline Phosphatase, ALT=Alanine Aminotransferase, AST=Aspartate Aminotransferase, BMI=Body Mass Index, BP=Blood Pressure, CEA=Carcinoembryonic Antigen, CML=Chronic Myeloid Leukaemia, Conc.=Concentration, CT=computed tomography, CVA=Cerebrovascular Accident, ESR=Erythrocyte Sedimentation Rate, FEV1=Forced Expiratory Volume in 1 second, FVC=Forced Vital Capacity, GGT=Gamma-Glutamyl Transpeptidase, Hx=history, IBD=Inflammatory Bowel Disease, IMD=Index of Multiple Deprivation, K=Potassium, LUQ pain=Left Upper Quadrant Pain, Na=Sodium, PR=Per Rectum, PSA=Prostate Specific Antigen, RUQ pain=Right Upper Quadrant Pain, TB=Tuberculosis, VC=Vital Capacity, YoB=year of birth^ | | | | | | | | | | | | | | | | | | | |  |
